# Supplementary material for: Progressive exercise versus best practice advice for adults aged 50 years or over after ankle fracture: the AFTER pilot randomised controlled trial
Source: BMJ Open. 2022 Nov 23;12(11):e059235. doi: 10.1136/bmjopen-2021-059235 (PMC9693648; doi:10.1136/bmjopen-2021-059235)
Supplement: Supplementary data [file bmjopen-2021-059235supp001.pdf]

Supplementary appendix

Table S1: *Health resource use at 3 months*

	Best practice advice		Progressive exercise		Total	
	n	%	n	%	n	%
**Pain increase**						
Doctor seen	0	0%	1	3.2%	1	1.6%
Painkillers	3	10%	2	6.5%	5	8.2%
Further pain	1	3.3%	0	0%	1	1.6%
**Condition worsened**						
Doctor seen	2	6.7%	1	3.2%	3	4.9%
Further treatment	1	3.3%	1	3.2%	2	3.3%
Further worsening	1	3.3%	0	0%	1	1.6%
**Prescription medications**						
Yes	7	23.3%	13	41.9%	20	32.8%
No	23	76.7%	18	58.1%	41	67.2%
**Over the counter medications**						
Yes	10	33.3%	7	22.6%	17	27.9%
No	20	66.7%	24	77.4%	44	72.1%
**NHS community services**						
GP visit	3	10%	2	6.5%	5	8.2%
GP telephone	0	0%	1	3.2%	1	1.6%
Practice nurse	0	0%	2	6.5%	2	3.3%
Physio at hospital	3	10%	1	3.2%	4	6.6%
Community health team	1	3.3%	0	0%	1	1.6%
Consultant for extra scan	1	3.3%	0	0%	1	1.6%
Other	1	3.3%	0	0%	1	1.6%
**NHS outpatient**						
Orthopaedic clinic	9	30%	6	19.4%	15	24.6%
Physiotherapy department	3	10%	0	0%	3	4.9%
X-ray	4	13.3%	2	6.5%	6	9.8%
Ultrasound	1	3.3%	0	0%	1	1.6%
A & E	2	6.7%	2	6.5%	4	6.6%
Other outpatient	4	13.3%	3	9.7%	7	11.5%
**NHS inpatient**						
Broken ankle	0	0%	1	3.2%	1	1.6%
**Private outpatient**						
Orthopaedic clinic	1	3.3%	1	3.2%	2	3.3%
Physiotherapy department	2	6.7%	1	3.2%	3	4.9%
Other outpatient	2	6.7%	0	0%	2	3.3%
**Expenses occurred**						
Yes	7	23.3%	19	61.3%	26	42.6%
No	15	50%	7	22.6%	22	36.1%
Unknown	8	26.7%	5	16.1%	13	21.3%
**Expenses breakdown**						
Travel and taxi fares	7	23.3%	6	19.4%	13	21.3%

	Best practice advice		Progressive exercise		Total	
	n	%	n	%	n	%
Parking	2	6.7%	2	6.5%	2	3.3%
Care costs	2	6.7%	2	6.5%	2	3.3%
Other	6	20%	4	12.9%	10	16.4%

Table S2: *Health resource use at 6 months*

	Best practice advice		Progressive exercise		Total	
	n	%	n	%	n	%
**Condition worsened**						
Doctor seen	1	3.3%	0	0%	1	1.6%
Further treatment	1	3.3%	0	0%	1	1.6%
**Prescription medications**						
Yes	4	13.3%	9	29.0%	13	21.3%
No	26	86.7%	22	71.0%	48	78.7%
**Over the counter medications**						
Yes	5	16.7%	2	6.5%	7	11.5%
No	25	83.3%	29	93.5%	54	88.5%
**NHS community services**						
GP visit	2	6.7%	3	9.7%	5	8.2%
Physio at home	0	0%	1	3.2%	1	1.6%
Physio at home	0	0%	1	3.2%	1	1.6%
**NHS outpatient**						
Orthopaedic clinic	0	0%	1	3.2%	1	1.6%
Physiotherapy department	1	3.3%	0	0%	1	1.6%
A & E	1	3.3%	0	0%	1	1.6%
**NHS inpatient**						
Unknown reason	0	0%	1	3.2%	1	1.6%
**Private outpatient**						
Physiotherapy department	2	6.7%	1	3.2%	3	4.9%
Other outpatient	2	6.7%	1	3.2%	3	4.9%
**Expenses occurred**						
Yes	2	6.7%	3	9.7%	5	8.2%
No	23	76.7%	23	74.2%	26	42.6%
Unknown	5	16.7%	5	16.1%	10	16.4%
**Expenses breakdown**						
Travel and taxi fares	0	0%	3	9.7%	3	4.9%
Parking	2	6.7%	0	0%	2	3.3%
Other	1	3.3%	4	12.9%	5	8.2%

Table S3: return to desired activities, including work, social life, and sport activities, walking aid use and distances at three months

	Best practice advice		Progressive exercise		Total	
	n	%	n	%	n	%
**Walking aids**						
Frame/rollator	1	3.3%	1	3.2%	2	3.3%
None	20	66.7%	20	64.5%	40	65.6%
One crutch	2	6.7%	1	3.2%	3	4.9%
One stick	3	10%	2	6.5%	5	8.2%
Two crutches	1	3.3%	1	3.2%	2	3.3%
Two sticks	0	0%	1	3.2%	1	1.6%
Missing	3	10%	5	16.1%	8	13.1%
**Walking distance possible**						
About the house	0	0%	2	6.5%	2	3.3%
Less than 100 metres	2	6.7%	2	6.5%	4	6.6%
Less than half a mile	4	13.3%	4	12.9%	8	13.1%
More than half a mile	21	70%	18	58.1%	39	63.9%
Missing	3	10%	5	16.1%	8	13.1%
**Recreational activities**						
Missing	5	16.7%	5	16.1%	10	16.4%
No difficulty	4	13.3%	8	25.8%	12	19.7%
Mild difficulty	11	36.7%	9	29%	20	32.8%
Moderate difficulty	4	13.3%	6	19.4%	10	16.4%
Severe difficulty	5	16.7%	0	0%	5	8.2%
Unable	1	3.3%	3	9.7%	4	6.6%
**Family/friend interference**						
Missing	4	13.3%	5	16.1%	9	14.8%
Not at all	12	40%	14	45.2%	26	42.6%
Slightly	8	26.7%	7	22.6%	15	24.6%
Moderately	2	6.7%	3	9.7%	5	8.2%
Quite a bit	3	10%	1	3.2%	4	6.6%
Extremely	1	3.3%	1	3.2%	2	3.3%
**Daily activities**						
Missing	4	13.3%	5	16.1%	9	14.8%
Not at all limited	11	36.7%	15	48.4%	26	42.6%
Slightly limited	7	23.3%	5	16.1%	12	19.7%
Moderately limited	7	23.3%	5	16.1%	12	19.7%
Very limited	1	3.3%	0	0%	1	1.6%
Extremely limited	0	0%	1	3.2%	1	1.6%
**Improvement[1]**						
-1	1	3.3%	1	3.2%	2	3.3%
-2	1	3.3%	0	0%	1	1.6%
-3	0	0%	1	3.2%	1	1.6%
0	0	0%	4	12.9%	4	6.6%
1	0	0%	2	6.5%	2	3.3%
2	1	3.3%	3	9.7%	4	6.6%
3	12	40%	6	19.4%	18	29.5%
4	6	20%	6	19.4%	12	19.7%
5	2	6.7%	3	9.7%	5	8.2%

	Best practice advice		Progressive exercise		Total	
	n	%	n	%	n	%
Missing	7	23.3%	5	16.1%	12	19.7%
**Doing Exercises**						
Missing	7	23.3%	6	19.4%	13	21.3%
No	3	10%	4	12.9%	7	11.5%
Yes	20	66.7%	21	67.7%	41	67.2%
**Exercise frequency (days per week)**						
Missing	10	33.3%	10	32.3%	20	32.8%
2	1	3.3%	0	0%	1	1.6%
3	3	10%	1	3.2%	4	6.6%
5	5	16.7%	5	16.1%	10	16.4%
6	2	6.7%	1	3.2%	3	4.9%
7	9	30%	14	45.2%	23	37.7%
**Employment**						
Missing	6	20%	5	16.1%	11	18%
No	15	50%	14	45.2%	29	47.5%
Yes	9	30%	12	38.7%	21	34.4%
**Time-off due to ankle injury**						
Missing	21	70%	20	64.5%	41	67.2%
No	4	13.3%	6	19.4%	10	16.4%
Yes	5	16.7%	5	16.1%	10	16.4%

[1] Scale ranges from -5 denoting 'very much worse' to 5 denoting 'completely recovered', and with 0 denoting 'unchanged'

Table S4: return to desired activities, including work, social life, and sport activities, walking aid use and distances at six months

	Best practice advice		Progressive exercise		Total	
	n	%	n	%	n	%
**Walking aids**						
Frame/rollator	1	3.3%	1	3.2%	2	3.3%
None	22	73.3%	24	77.4%	46	75.4%
One stick	4	13.3%	2	6.5%	6	9.8%
Two crutches	0	0%	1	3.2%	1	1.6%
Missing	3	10%	3	9.7%	6	9.8%
**Walking distance possible**						
Bedbound	0	0%	1	3.2%	1	1.6%
About the house	0	0%	1	3.2%	1	1.6%
Less than 100 metres	3	10%	1	3.2%	4	6.6%
Less than half a mile	2	6.7%	2	6.5%	4	6.6%
More than half a mile	22	73.3%	23	74.2%	45	73.8%
Missing	3	10%	3	9.7%	6	9.8%
**Recreational activities**						
Missing	3	10%	4	12.9%	7	11.5%

	Best practice advice		Progressive exercise		Total	
	n	%	n	%	n	%
No difficulty	13	43.3%	18	58.1%	31	50.8%
Mild difficulty	9	30%	6	19.4%	15	24.6%
Moderate difficulty	2	6.7%	2	6.5%	4	6.6%
Severe difficulty	2	6.7%	0	0%	2	3.3%
Unable	1	3.3%	1	3.2%	2	3.3%
**Family/friend interference**						
Missing	3	10%	4	12.9%	7	11.5%
Not at all	19	63.3%	21	67.7%	40	65.6%
Slightly	6	20%	2	6.5%	8	13.1%
Moderately	1	3.3%	1	3.2%	2	3.3%
Quite a bit	0	0%	2	6.5%	2	3.3%
Extremely	1	3.3%	1	3.2%	2	3.3%
**Daily activities**						
Missing	3	10%	4	12.9%	7	11.5%
Not at all limited	17	56.7%	20	64.5%	37	60.7%
Slightly limited	8	26.7%	5	16.1%	13	21.3%
Moderately limited	2	6.7%	0	0%	2	3.3%
Very limited	0	0%	1	3.2%	1	1.6%
Extremely limited	0	0%	1	3.2%	1	1.6%
**Improvement[1]**						
-1	0	0%	3	9.7%	3	4.9%
-3	2	6.7%	0	0%	2	3.3%
-4	0	0%	1	3.2%	1	1.6%
Missing	3	10%	4	12.9%	7	11.5%
0	1	3.3%	1	3.2%	2	3.3%
2	1	3.3%	3	9.7%	4	6.6%
3	4	13.3%	2	6.5%	6	9.8%
4	13	43.3%	9	29%	22	36.1%
5	6	20%	8	25.8%	14	23%
**Doping Exercises**						
Missing	4	13.3%	5	16.1%	9	14.8%
No	12	40%	7	22.6%	19	31.1%
Yes	14	46.7%	19	61.3%	33	54.1%
**Exercise frequency (days per week)**						
Missing	17	56.7%	12	38.7%	29	47.5%
1	0	0%	1	3.2%	1	1.6%
2	1	3.3%	1	3.2%	2	3.3%
3	2	6.7%	6	19.4%	8	13.1%
4	2	6.7%	3	9.7%	5	8.2%
5	2	6.7%	3	9.7%	5	8.2%
6	0	0%	1	3.2%	1	1.6%
7	6	20%	4	12.9%	10	16.4%
**Employment**						
Missing	2	6.7%	4	12.9%	6	9.8%
No	18	60%	14	45.2%	32	52.5%
Yes	10	33.3%	13	41.9%	23	37.7%
**Time-off due to ankle injury**						
Missing	20	66.7%	18	58.1%	38	62.3%

	Best practice advice		Progressive exercise		Total	
	**n**	**%**	**n**	**%**	**n**	**%**
No	9	30%	11	35.5%	20	32.8%
Yes	1	3.3%	2	6.5%	3	4.9%

[1] Scale ranges from -5 denoting ‘very much worse’ to 5 denoting ‘completely recovered’, and with 0 denoting ‘unchanged’

Table S5: *Adverse events summarised at 3 and 6 months*

	**Best practice advice**		**Progressive exercise**		**Total**	
	**n**	**%**	**n**	**%**	**n**	**%**
**3 Months**						
**Recent falls (last 3 months)**						
No	22	73.3%	22	71%	44	72.1%
Yes	4	13.3%	4	12.9%	8	13.1%
Missing	4	13.3%	5	16.1%	9	14.8%
**Number of recent falls**						
0	26	86.7%	27	87.1%	53	86.9%
1	2	6.7%	3	9.7%	5	8.2%
2	1	3.3%	0	0%	1	1.6%
3	1	3.3%	0	0%	1	1.6%
6	0	0%	1	3.2%	1	1.6%
**Broken bones (from recent fall, if applicable)**						
Yes	0	0%	0	0%	0	0%
No	4	13.3%	4	12.9%	8	13.1%
**Pain increase**						
Missing	6	20%	8	25.8%	14	23%
No	21	70%	21	67.7%	42	68.9%
Yes	3	10%	2	6.5%	5	8.2%
**Condition worsened**						
Missing	5	16.7%	8	25.8%	13	21.3%
No	22	73.3%	21	67.7%	43	70.5%
Yes	3	10%	2	6.5%	5	8.2%
**6 Months**						
**Recent falls (last 3 months)**						
No	23	76.7%	25	80.6%	48	78.7%
Yes	3	10%	2	6.5%	5	8.2%
Missing	4	13.3%	4	12.9%	8	13.1%
**Number of recent falls**						
0	27	90%	29	93.5%	56	91.8%
1	2	6.7%	1	3.2%	3	4.9%
2	1	3.3%	0	0%	1	1.6%
5	0	0%	1	3.2%	1	1.6%
**Broken bones (from recent fall)**						
Yes	1	3.3%	0	0%	1	1.6%
No	2	6.7%	2	6.5%	4	6.6%
**Pain increase**						
Missing	3	10%	5	16.1%	8	13.1%
No	27	90%	26	83.9%	53	86.9%
Yes	0	0%	0	0%	0	0%
**Condition worsened**						
Missing	3	10%	6	19.4%	9	14.8%
No	26	86.7%	25	80.6%	51	83.6%
Yes	1	3.3%	0	0%	1	1.6%
