# Supplementary material for: Progressive exercise versus best practice advice for adults aged 50 years or over after ankle fracture: the AFTER pilot randomised controlled trial
Source: BMJ Open. 2022 Nov 23;12(11):e059235. doi: 10.1136/bmjopen-2021-059235 (PMC9693648; doi:10.1136/bmjopen-2021-059235)
Supplement: Author's manuscript [file bmjopen-2021-059235.draft_revisions.pdf]

BMJ Open

BMJ Open is committed to open peer review. As part of this commitment we make the peer review history of every article we publish publicly available.

When an article is published we post the peer reviewers' comments and the authors' responses online. We also post the versions of the paper that were used during peer review. These are the versions that the peer review comments apply to.

The versions of the paper that follow are the versions that were submitted during the peer review process. They are not the versions of record or the final published versions. They should not be cited or distributed as the published version of this manuscript.

BMJ Open is an open access journal and the full, final, typeset and author-corrected version of record of the manuscript is available on our site with no access controls, subscription charges or pay-per-view fees (http://bmjopen.bmj.com).

If you have any questions on BMJ Open's open peer review process please email
info.bmjopen@bmj.com

BMJ Open

Progressive exercise versus best practice advice for adults aged 50 years or over after ankle fracture: the AFTER pilot randomised controlled trial

Journal:	*BMJ Open*
Manuscript ID	bmjopen-2021-059235
Article Type:	Original research
Date Submitted by the Author:	15-Nov-2021
Complete List of Authors:	Keene, David; University of Oxford, Oxford Trauma and Emergency Care, Nuffield Department of Orthopaedics, Rheumatology and Musculoskeletal Sciences<br>Costa, Matthew; University of Oxford, Nuffield Department of Orthopaedics, Rheumatology, and Musculoskeletal Sciences<br>Peckham, Nicholas; University of Oxford<br>Tutton, Elizabeth; University of Oxford, NDORMS<br>Barber, Vicki; University of Oxford Nuffield Department of Orthopaedics Rheumatology and Musculoskeletal Sciences, Oxford Clinical Trials Unit<br>Dutton, Susan; University of Oxford, CSM<br>Hopewell, Sally; University of Oxford, Nuffield Department of Orthopaedics, Rheumatology and Musculoskeletal Sciences<br>Redmond, Anthony; University of Leeds, Leeds Institute of Rheumatology and Musculoskeletal Medicine<br>Willett, Keith; University of Oxford, Orthopaedic Trauma Surgery<br>Lamb, Sarah; University of Exeter, College of Medicine and Health; University of Oxford, NDORMS<br>trial collabororators, AFTER ; trial collabororators
Keywords:	Foot & ankle < ORTHOPAEDIC & TRAUMA SURGERY, Rehabilitation medicine < INTERNAL MEDICINE, Clinical trials < THERAPEUTICS

SCHOLARONE™
Manuscripts

BMJ

I, the Submitting Author has the right to grant and does grant on behalf of all authors of the Work (as defined in the below author licence), an exclusive licence and/or a non-exclusive licence for contributions from authors who are: i) UK Crown employees; ii) where BMJ has agreed a CC-BY licence shall apply, and/or iii) in accordance with the terms applicable for US Federal Government officers or employees acting as part of their official duties; on a worldwide, perpetual, irrevocable, royalty-free basis to BMJ Publishing Group Ltd ("BMJ") its licensees and where the relevant Journal is co-owned by BMJ to the co-owners of the Journal, to publish the Work in this journal and any other BMJ products and to exploit all rights, as set out in our licence.

The Submitting Author accepts and understands that any supply made under these terms is made by BMJ to the Submitting Author unless you are acting as an employee on behalf of your employer or a postgraduate student of an affiliated institution which is paying any applicable article publishing charge ("APC") for Open Access articles. Where the Submitting Author wishes to make the Work available on an Open Access basis (and intends to pay the relevant APC), the terms of reuse of such Open Access shall be governed by a Creative Commons licence – details of these licences and which Creative Commons licence will apply to this Work are set out in our licence referred to above.

Other than as permitted in any relevant BMJ Author's Self Archiving Policies, I confirm this Work has not been accepted for publication elsewhere, is not being considered for publication elsewhere and does not duplicate material already published. I confirm all authors consent to publication of this Work and authorise the granting of this licence.

For peer review only

Progressive exercise versus best practice advice for adults aged 50 years or over after ankle fracture: the AFTER pilot randomised controlled trial

David J. Keene[1*]
Matthew L. Costa[1]
Nicholas Peckham[2]
Elizabeth Tutton[1,3]
Vicki S. Barber[2]
Susan J. Dutton[2]
Sally Hopewell[2]
Anthony C. Redmond[4]
Keith Willett[1,2]
Sarah E. Lamb[5]
on behalf of the AFTER trial collaborators

[1] Oxford Trauma and Emergency Care, Nuffield Department of Orthopaedics, Rheumatology and Musculoskeletal Sciences, University of Oxford, Oxford, UK
[2] Oxford Clinical Trials Research Unit (OCTRU), University of Oxford, Oxford, UK
[3] Oxford University Hospitals NHS Foundation Trust, Oxford, UK
[4] Leeds Institute of Rheumatic and Musculoskeletal Medicine, University of Leeds, Leeds, UK
[5] College of Medicine and Health, University of Exeter, Exeter. UK

Word count:

*corresponding author
Dr David J. Keene
Kadoorie Centre, Level 3, John Radcliffe Hospital, OX3 9DU
david.keene@ndorms.ox.ac.uk

For peer review only - http://bmjopen.bmj.com/site/about/guidelines.xhtml

ABSTRACT

Objective

The aim of the AFTER study, an external multicentre pilot randomised controlled trial, was to assess the feasibility of a definitive trial comparing rehabilitation approaches for adults aged 50 years and over after an ankle fracture.

Setting

Five UK National Health Service hospitals

Participants

Participants were aged 50 years and over with an ankle fracture requiring surgery or non-operative management by immobilisation for at least 4 weeks.

Interventions

Participants were allocated 1:1 via a central web-based randomisation system to: i) best-practice advice (one session of physiotherapy, up to two optional additional advice sessions) or ii) progressive exercise (up to six sessions of physiotherapy).

Primary outcome measures:

Feasibility: i) participation rate ii) intervention adherence iii) retention.

Results

Sixty-one of 112 (54%) eligible participants participated, exceeding progression criteria for participation of 25%. Recruitment progression criteria was 1.5 participants per site per month, 1.4 was observed. Intervention sessions were fully or partially delivered for 28/30 (93%) of best practice advice and 28/31 (90%) of progressive exercise participants, exceeding the 85% progression criteria. For those providing follow-up data, the proportion of participants performing home exercises in the best practice advice and progressive exercise groups at three months was 20/23 (87%) and 21/25 (84%) respectively. Mean time from injury to starting physiotherapy was 74.1 days (95%CI 53.9 to 94.1 days) for best practice advice and 72.7 days (95%CI 54.7 to 88.9) for progressive exercise. Follow-up rate (6 month OMAS scores) was 28/30 (93%) for the best practice advice group and 26/31 (84%) in the progressive exercise group, an overall follow-up rate of 89%.

For peer review only - http://bmjopen.bmj.com/site/about/guidelines.xhtml

Conclusions

This pilot RCT demonstrated a definitive trial would be feasible. The main issues to address for a definitive trial are intervention modifications to enable earlier provision of rehabilitation and ensuring similar rates of follow-up in each group.

Trial registration: ISRCTN16612336

Keywords

Ankle Fractures, Clinical Trial, Geriatrics, Exercise Therapy

Strengths and limitations of this study

- Our pilot RCT demonstrated that a definitive trial comparing a best practice advice versus a supervised progressive exercise intervention for adults aged 50 years and over after ankle fracture is feasible.
- All feasibility success criteria were met, apart from a minor difference in the recruitment rate per site-month that would need to be factored into planning a definitive trial.
- Follow-up rates differed between the groups, ensuring similar rates in a definitive trial is therefore critical.
- A limitation of the study, common to all pilot trials, is that with the necessarily limited number of centres, there remains some uncertainty regarding the feasibility of a trial across a much larger number of centres.

For peer review only

INTRODUCTION

Ankle fractures are very common, accounting for 9% of all fractures managed in secondary care.[1] In the UK, incidence of these fractures is highest in people aged 50 years and over, peaking at 16 per 10,000 person-years in women aged 60 to 70.[2] As the population ages, a three-fold increase in these fractures is projected over the next two decades.[3] The mechanism of injury for people aged over 50 is commonly a fall from standing height, which are defined as fragility fractures.[4]

Treatments for ankle fractures range from conservative plaster casts or boots to surgical fixation. The AIM trial found that regardless of the initial fracture management, at 6 months reduced ankle function and walking abnormalities are substantial.[5 6] Participants reported an average 30% loss of pre-injury ankle function. Function is poor due to pain, reduced joint motion,[7] lower limb muscle strength deficits,[8] gait abnormalities,[9] and resultant mobility limitations.[7 10]

Weight bearing and ankle movement restrictions are usually removed by the orthopaedic team 6 weeks after injury. A Cochrane review[11] of ankle fracture rehabilitation concluded that there was insufficient evidence to support traditional physiotherapy interventions targeting ankle joint and muscle impairments, such as stretching,[12] manual therapy,[13] and exercise.[14] Updating the Cochrane review searches in MEDLINE and EMBASE, identified another multicentre trial by Moseley and colleagues.[15] They found no differences in self-reported lower limb function or quality of life between supervised exercise and a one-off advice session for adults with ankle fractures treated surgically and conservatively. Physiotherapists delivered both interventions face-to-face and the mean age of participants was 42 years.

Physiotherapy interventions tested to date have not incorporated features of advice and exercise programmes for older adults used in other rehabilitation areas. Issues such as persistent poor balance in older adults require attention.[16] The Ankle Fracture Treatment: Enhancing Rehabilitation (AFTER) study was a pilot randomised controlled trial to assess

For peer review only

feasibility of a definitive trial comparing best-practice advice with a supervised progressive exercise programme for adults aged 50 years and over after ankle fracture.

Feasibility Objectives

The main objectives of the pilot trial were to:

- Assess patient engagement with the trial, measured by the participation rate of those eligible.
- Establish whether the interventions were acceptable to participants and therapists, assessed by intervention adherence levels (and participant interviews and a therapist focus group – reported separately).
- Determine patient retention, measured by the proportion of patients providing outcome data at 6 months
- Assess the acceptability of measuring outcomes at 3 and 6 months post randomisation.

METHODS

Study design

A multi-centre pilot parallel-group RCT. Participants were allocated to either: i) best-practice advice (one session of face-to-face advice delivered by a physiotherapist, with up to two further optional advice sessions as deemed required by the physiotherapist) or ii) progressive exercise (up to six sessions of individual face-to-face physiotherapy). The pilot trial protocol has been published.[17] An embedded qualitative study including patients and staff was also completed and will be reported separately.

Setting

Recruitment was from five NHS hospitals. Participants were identified while inpatients or when attending fracture clinics, where they were screened and given study information.

Study participants

For peer review only - http://bmjopen.bmj.com/site/about/guidelines.xhtml

The target population was adults aged 50 years as bone density reduces at this age and there is an increased risk of fragility fractures.[18] There is also a bimodal age distribution for ankle fractures, with peak incidences being for young adult males, and highest for females aged 50 years and over.[2] Participants were attending NHS services to manage ankle fractures that either required definitive management with surgical, or non-surgical management of ankle immobilisation for at least 4 weeks.

Patients were excluded if they:

- were unable to adhere to trial procedures or complete questionnaires
- did not have capacity to consent to study participation
- were not ambulatory before the injury
- were considered inappropriate for referral to physiotherapy by the clinician
- could not attend outpatient physiotherapy at a participating centre
- had serious concomitant disease (such as terminal illness)
- had bilateral lower limb fractures
- had an ipsilateral concurrent Pilon fracture
- had open fracture wounds, external fixation, or substantial skin loss or grafts that would limit ankle or lower leg exercise

Randomisation

Participants were randomised 1:1 to the intervention groups using the centralised computer randomisation service provided by the Oxford Clinical Trials Research Unit. The site's research facilitator undertook randomisation directly themselves or contacted the study office over the telephone to access the system on their behalf. Randomisation was computer-generated and stratified by centre and initial fracture management (surgery or non-surgical) using variable block sizes (2 and 4).

Blinding

Physiotherapists delivering the intervention and study participants were told the treatment allocation. Where possible, physiotherapists or trained research associates independent of the clinical team collected the objective outcome measures at the 6-month follow-up time point.

For peer review only

For peer review only - http://bmjopen.bmj.com/site/about/guidelines.xhtml

Interventions

The best-practice advice or progressive exercise sessions were given when the participant could mobilise with unrestricted weight-bearing and do ankle exercises as guided by their surgeon or physiotherapist. We anticipated that this would usually be around 6-8 weeks post-injury. Both interventions were delivered face-to-face and one-to-one by a physiotherapist. The interventions are outlined in Table 1. In summary:

Best-practice advice

The best-practice advice intervention included a 20 to 60 minute session (depending on local service provision) of assessment, education, reassurance, and detailed self-management advice on ankle exercises, gait training, stair climbing, walking aid advice, and basic balance exercises. An advice booklet provided a summary of key information.

Up to two further advice sessions were optional for participants having difficulties with self-management or the exercises. The physiotherapist's role was to re-assess and re-enforce self-management advice. During the initial stage of recruitment these additional sessions involved a telephone call or an additional face-to-face contact. In the latter stage of recruitment (after 41 participants had been recruited), these sessions were to be offered as telephone consultations only. This change was introduced to assess whether a greater focus on the single face-to-face session was acceptable to patients and physiotherapists. Use of additional sessions were recorded and monitored.

Progressive exercise

A physiotherapist instructed participants and supervised their progressive exercise programme in up to six sessions over 16 weeks. This period aimed to allow sufficient time for neuromuscular adaptation to exercise.[19] The programme could end early if all rehabilitation goals were achieved in under six sessions. The first session was 20-60 minutes and the rest were up to 30 minutes, consistent with the duration of physiotherapy sessions in the NHS. Physiotherapists provided assessment, advice, education about progressing recovery, gait training, walking aid instruction, and an information booklet.

For peer review only - http://bmjopen.bmj.com/site/about/guidelines.xhtml

The programme was highly structured but calibrated for each individual. All participants received a core set of functional lower limb resistance exercises in line with the evidence for improving muscular strength and power in older persons' rehabilitation.[20] The programme included supervised gait training to target major walking difficulties after ankle fracture. Balance exercises were included in the programme and introduced once the participant was able to weight bear sufficiently to perform these. Exercises were practised in the clinic but conducted at home with the aim of achieving an effective dose. Based on the participant's functional goals, exercises were progressed to make them task-specific, for example walking on uneven surfaces or slopes, climbing stairs, or jogging. Participants received an exercise planner and diary. Exercise progression was based on evidence-based guidelines[21] but individualised by progressing and regressing the volume and load in line with each participant's capabilities and preferences.

The progressive exercise intervention used simple health behaviour change techniques to optimise adherence to home exercise.[22] Participants were asked to identify their goals following usual physiotherapy practice and, with the treating therapist's help, write an action plan for where and when they would perform their home exercises and a contingency plan for managing difficulties. Therapists were trained to focus on helping participants identify barriers to exercise and becoming more physically active post-injury, and facilitating problem-solving. A high-quality information booklet was developed by the AFTER study team with Patient and Public Involvement representatives and provided to participants.

Training and monitoring of intervention delivery

Treating therapists were trained in a face-to-face session of up to four hours and provided with written materials on the theory and practical delivery of the interventions. The same therapists were trained in both interventions. Intervention delivery was recorded in logs for each contact with participants to monitor intervention fidelity and enable ongoing feedback to manage contamination. Local site Principle Investigators were also responsible for monitoring intervention delivery.

For peer review only - http://bmjopen.bmj.com/site/about/guidelines.xhtml

Best practice advice delivery adherence was defined as attendance of at least one physiotherapy session. For progressive exercise delivery adherence was defined as attending all six sessions, or if they were discharged by the physiotherapist as treatment completed (as marked on treatment log), or discharged by the physiotherapist following a patient-initiated follow-up period with no further contact, or if they were referred on for further investigations/treatment. Partial adherence for progressive exercise was attending at least one session but not meeting the outlined criteria for full adherence. Additional sessions were recorded for any best practice advice sessions after the main first sessions and for progressive exercise it as any sessions in addition to the six sessions.

Concomitant care

Other aspects of health and social care continued as normal. Additional treatments, including contact with their GP or other health professionals, were recorded in participant follow-up questionnaires.

Outcome measures

Feasibility success criteria

The main aim of this pilot RCT was to determine the feasibility of a future definitive trial.[23] The main uncertainty was whether patients find it acceptable to be randomised to different types of rehabilitation provision. To determine the feasibility of a definitive RCT, the success criteria were:

- A study participation rate of at least 25% of those eligible, to indicate acceptability and generalisability
- At least 48 eligible patients across at least three sites agree to participate over a maximum of 18 months
- At least 85% of participants completed study intervention sessions
- At least 80% of participants attended study follow-up at 6 months, defined as the proportion of participants who provided OMAS scores at 6 months

Outcomes

For peer review only

Participants were followed up 3 months after randomisation with a postal questionnaire and 6 months after randomisation face-to-face at the hospital. They were offered telephone or postal follow-up if they were unable to attend the 6-month follow-up.

Patient-reported outcomes at 3 and 6 months were:

- ankle-related symptoms and function: Olerud and Molander Ankle Score (0-100, higher scores better)[24]
- lower-limb function limitations: Lower Extremity Functional Scale (0-100, higher scores better)[25]
- pain: visual analogue scale (VAS), 0-100 scale, higher scores worse
- health-related quality of life: EQ-5D-5L score.[26] The EQ-5D health status scale is mapped onto the EQ-5D-3L valuation set using the Crosswalk Index Value Calculator.[27] The scale from this set ranges from -0.594, indicating the worst possible health state, to 1.0 and is anchored at 0 [death] and 1.0 full health]. A respondent's EQ-VAS gives self-rated health on a scale where the endpoints are labelled from 'worst imaginable health state' (0) to 'best imaginable health state' (100) .
- fear of falls: Falls Efficacy Scale-International (short version).[28] The overall scores range from 7 (no concern about falling) to a maximum of 28 (severe concern about falling).
- self-efficacy: self-efficacy exercise score.[29] Overall scores range 0 to 90, with higher scores indicating greater confidence to exercise.
- self-reported return to desired activities, including work, social life, and sport activities
- walking aid use and distances
- exercise adherence (self-reported exercise frequency)
- health resource use (including additional out of trial physiotherapy).

At 6-month follow-up, a blinded outcome assessor collected objective measures of ankle function and physical performance:

- ankle joint range: hand-held goniometry[30]

For peer review only - http://bmjopen.bmj.com/site/about/guidelines.xhtml

- muscle strength: hand-held dynamometry (Lafayette Manual Muscle Test System, Lafayette Instrument, IN, USA) of ankle dorsi/plantarflexion using a 'make' approach (working up to a maximal contraction over a maximum of 5 seconds and without pushing into pain and the assessor maintaining position of the device).[31] Participants were measured 3 times and had at least 10 seconds rest between attempts.
- mobility and balance: Short Physical Performance Battery (SPPB).[32] The test involves physical tests of balance, walking speed, and repeated rises from a chair.

Adverse events

Expected general side effects of any exercise, such as delayed-onset muscle soreness and temporary increases in pain of less than a week, were not recorded as adverse events. Pain increases of more than a week were recorded in patient-reported questionnaires. Any exacerbations of other medical conditions during exercise or exercise-related injurious falls could also be recorded in patient-reported questionnaires, or by the site investigators if they become aware of such an event.

Sample size

The main feasibility objective and therefore the basis of the sample size estimate was participant recruitment per centre. The target sample size was a minimum of 48 participants in at least three centres over a maximum of 18 months. The sample size was based on recruiting 1.5 participants per month per site. An amendment to the protocol during recruitment enabled the recruitment to continue up to 60 participants so that more feasibility data could be collected after the changes to the best practice advice intervention outlined above.

Statistical analysis

Baseline characteristics and outcomes were reported using descriptive statistics using mean and standard deviation (or median and interquartile range if non-normally distributed), and minimum and maximum, for continuous variables and number and percentage of patients in each group for binary or categorical variables. The study was not powered for formal hypothesis testing between the treatment arms.

For peer review only - http://bmjopen.bmj.com/site/about/guidelines.xhtml

Research ethics approval

The study was approved by the South Central – Hampshire B Research Ethics Committee (Ref: 18/SC/0281) on 2nd July 2018 and the Health Research Authority and was prospectively registered (ISRCTN16612336).

We used the Consolidated Standards of Reporting Trials (CONSORT) guidelines for pilot and feasibility trials[33 34] and the Template for Intervention Description and Replication (TIDieR) statement to report the study.[35]

Patient and Public Involvement

The intervention development and study materials were supported by a patient and public involvement group, who were also involved in interpreting the study findings and definitive trial protocol design.

RESULTS

Screening, recruitment and baseline characteristics

Patient enrolment began in October 2018 and was completed in August 2019 when the sample size was reached. Of the 226 patients screened, 112 were eligible, and 61 were recruited and randomised (see Figure 1). The main reason screened patients were not eligible was that they were unable to attend outpatient physiotherapy at a participating centre (41/114, 36%). Of the 34 patients who declined participation, the main reason provided was that they did not want to return to the participating centre for the study due to practical difficulties such as transport and parking (8/34, 24%). The revised sample size target of 60 was exceeded as one participant had consented to participation and randomised before closure of the randomisation system.

Baseline characteristics are summarised in Table 2. Participants had a mean age of 63.5 (SD 8.9) years, and 42/61 (69%) were female. 41/61 (67%) had surgery and the remainder (20/61, 33%) had non-surgical treatment for their ankle fracture.

Feasibility outcomes

For peer review only - http://bmjopen.bmj.com/site/about/guidelines.xhtml

Patient engagement with the trial: The final recruitment rate of eligible participants was 54%. The overall recruitment rate per month per site was 1.4 participants.

Intervention adherence levels: The mean time from randomisation to having the first physiotherapy session was 39.2 days (95% CI 27.3 to 55.3) for best practice advice and 38.2 (95% CI 29.8 to 48.1) for progressive exercise. From injury to starting physiotherapy it was 74.1 days (95% CI 53.9 to 94.1 days) for best practice advice and 72.7 days (95% CI 54.7 to 88.9) for progressive exercise.

The intervention sessions were fully or partially delivered in 28/30 (93%) best practice advice and 28/31 (90%) progressive exercise participants (see Table 3). Overall the levels of fidelity in delivery of the intervention components was high, see Table 4. The median number of physiotherapy sessions for best practice advice was 1 (IQR 1,2) and for progressive exercise it was 5 (IQR 3, 6).

For the progressive exercise programme, there was evidence in treatment logs of exercise prescription progression (exercise type, volume or load) across sessions for 21/28 (75%) of participants. Exercises were not progressed for one participant and none regressed over the sessions. It was not possible to assess for six participants due to insufficient exercise prescription data.

At 3 months post-randomisation, the proportion of participants performing home exercises out of those providing data in the best practice advice and progressive exercise groups was 20/23 (87%) and 21/25 (84%) respectively. At six months this reduced to 14/26 (54%) in the best practice advice group and 19/26 (73%) in the progressive exercise group.

Of the 28 participants allocated to best practice advice group that received at least the first session, eight (29%) had further contacts with their physiotherapist. Four participants that had an additional physiotherapy session (4/28, 14%) had clinical reasons for having a further face to face session (no more than two provided) due to issues with pain and concerns cited regarding progress and discolouration of the ankle. The other four participants had issues with appointment timing and scheduling, two participants needed extra face-to-face sessions as they had their first sessions scheduled too early (before they were permitted to

For peer review only - http://bmjopen.bmj.com/site/about/guidelines.xhtml

weight bear), another participant arrived too late for their appointment so had to come back to complete delivery, and one participant was initially seen in physiotherapy in error and had a usual care physiotherapy appointment, they had an extra session to receive the best practice advice session. Of the five participants who had additional contact with their physiotherapist after the protocol change (see Methods), two were by telephone, and three were face-to-face. One participant in the progressive exercise group had a further contact in addition to the six physiotherapy sessions. Three participants in each group reported receiving sessions of physiotherapy outside the trial (see health resource use data in the supplementary appendix).

Patient retention and outcome measure data collection: The patient-reported and physical outcome assessments are reported in Table 6. Follow up rate for OMAS scores at 6 months was 28/30 (93%) for the best practice advice group and 26/31 (84%) in the progressive exercise group, an overall follow-up rate of 89%. Completion rates for the objective outcome assessments at 6 months were lower than for the OMAS (e.g. 47/61 [77%] for SPPB). Data on return to desired activities, including work, social life, and sport activities, walking aid use and distances, and health resource use are reported in the supplementary appendix.

Adverse events: No serious adverse events were reported. Increases in pain of more than one week were reported by three participants in the best practice advice group and two in the progressive exercise group, all at the three month timepoint. One participant in the progressive exercise group consulted a physician due to this symptom increase. No increases in pain were reported at 6 months. Five participants reported a worsening of a medical condition at three months (three in the best practice advice group and two in the progressive exercise group). Of these reports, 3/5 (60%) sought medical attention as a result. One participant in the best practice group reported a worsening of a medical condition at six months and saw a physician.

A summary of the pre-specified feasibility success criteria and the respective outcomes are shown in Table 5.

For peer review only - http://bmjopen.bmj.com/site/about/guidelines.xhtml

DISCUSSION

Our pilot RCT demonstrated that a definitive trial comparing a best practice advice versus a supervised progressive exercise intervention for adults aged 50 years and over after ankle fracture is feasible. All feasibility success criteria were met, apart from a minor difference in the recruitment rate per site-month that would need to be factored into planning a definitive trial. Additionally, ensuring similar rates of follow-up in each intervention group is critical.

The main areas of refinement for the definitive RCT indicated by the pilot related to the interventions. Most patients had to wait several weeks after removal of the cast/splint for their first physiotherapy session. It would therefore be ideal for the early rehabilitation advice to start at the point where the cast or splint are removed rather than having to wait for a separate outpatient physiotherapy appointment. The most common reason participants did not want to participate was the requirement to attend the hospital for appointments. With the now widespread uptake of videoconferencing to deliver physiotherapy in the UK, a future trial where this is available as an option could make the trial more inclusive.

In the AFTER pilot RCT the we observed that in the best practice advice group about two in three participants had a single session of advice and did not use the optional additional sessions. Half of the additional sessions had clinical reasons, the remainder had issues with appointment timing and scheduling. The clinical reasons provided for the additional session seemed to relate to concerns regarding progress and symptoms that were concerning but anticipated after ankle fracture. The advice intervention could be improved with further information on symptoms and rate of progress to help address these early concerns. The majority of participants managed with a single session of advice without initiating the optional extra sessions. There was also minimal and balanced use of physiotherapy outside the trial over the six months (three participants in each intervention group, 6/61 [10%] in total). This contrasts to the previously largest trial of advice versus supervised rehabilitation, the EXACT trial,[15] where 39/108 (36%) of the advice group and 15/106 (14%) of the

For peer review only - http://bmjopen.bmj.com/site/about/guidelines.xhtml

physiotherapy supervised rehabilitation group had additional physiotherapy outside the trial, 54/214 (25%) in total.

In comparison to the outcome measure completion rates for patient-reported outcomes, there were lower rates of physical outcome measures. Given the greater extent of missing outcome data and the extra clinical and participant burden, and the context of trying to reduce hospital visits since the start of the COVID-19 pandemic, use of remote follow-up with questionnaires only is an important option for the definitive RCT.

We asked participants to self-report adverse events as one of the interventions had a much greater level of contact with a health professional and it was anticipated that there would be few serious adverse events. However, the validity of the relatedness of adverse events to the participants rehabilitation is difficult to assess when dependant on self-report. The recruiting centres could also report adverse events, this aimed to identify the more serious treatment-related complications. Overall, the challenging progressive exercise programme appeared to be generally well tolerated as there were few adverse events, no serious adverse events, and those events reported were similar between the intervention groups.

A limitation of the study, common to all pilot trials is that with the necessarily limited number of centres, there remains some uncertainty regarding the feasibility of a trial across a much larger number of centres. We attempted to mitigate this issue by running the trial at a range of trauma units and major trauma centres. These centres had varied geographical settings, diverse populations, and a range of levels of experience in running multicentre trauma trials. Physiotherapists were trained in both interventions so there was a potential risk of contamination. Although treatment logs indicated good levels of intervention fidelity, more onsite observation and/or recording of interventions would have enabled a more robust assessment of fidelity. Training separate physiotherapists in each intervention would also be advisable where it is practical to do so to further reduce the risk of contamination.

The evidence from this pilot RCT demonstrates that with refinement, a definitive trial would be feasible. This feasibility assessment is valuable, a full trial is deliverable and acceptable to patients and health care professionals and has subsequently been funded. Investment in

For peer review only - http://bmjopen.bmj.com/site/about/guidelines.xhtml

pilot/feasibility studies have been shown to be a good investment from a funders perspective, with an analysis from a UK-based research funder clearly demonstrating value in terms of reducing the historic trend of trials failing to be delivered.[36]

For peer review only

For peer review only - http://bmjopen.bmj.com/site/about/guidelines.xhtml

Acknowledgements

The AFTER study collaborating Principal Investigators were:

Hannah Perkins (Oxford University Hospitals NHS Foundation Trust)

Carol McCrum (East Sussex Healthcare NHS Trust)

Jacky Jones (Guy's and St Thomas' NHS Foundation Trust)

Carey McClellan and Naomi Chalk (University Hospitals NHS Foundation Trust)

Fiona Cowell (Royal Liverpool and Broadgreen University Hospital NHS Trust)

This study will be/has been conducted as part of the portfolio of trials in the registered UKCRC Oxford Clinical Trials Research Unit (OCTRU) at the University of Oxford. It will follow/has followed their Standard Operating Procedures ensuring compliance with the principles of Good Clinical Practice and the Declaration of Helsinki and any applicable regulatory requirements. Special thanks to Nicola Kenealy, Molly Glaze, Gemma Greenall, Rebecca MacLean, Emma Haines, Lucy Eldridge, Juul Achten, Kylea Draper, Elisa Basso, Amrita Athwal and the wider team at the OCTRU and all the physiotherapists, surgeons and research associates at the recruiting centres. Thanks also to Colin Forde for contributing to intervention development.

Author contributions

DJK is the chief investigator and conceived and designed the study, was awarded the funding, and drafted the manuscript. MLC, ET, SH, VSB, SJD, ACR, KW and SEL contributed to study design and provided specific content and edited the manuscript. SJD oversaw the statistical aspects of the study. VSB provided trial management oversight. All authors have reviewed and approved the manuscript.

Funding statement

This report is independent research supported by the National Institute for Health Research (NIHR Post-Doctoral Fellowship, Dr David Keene, PDF-2016-09-056). The report was supported by the NIHR Biomedical Research Centre, Oxford. Professor Lamb received funding from the NIHR Collaboration for Leadership in Applied Health Research and Care Oxford at Oxford Health NHS Foundation Trust.

For peer review only - http://bmjopen.bmj.com/site/about/guidelines.xhtml

For peer review only

Disclaimer

The views expressed in this publication are those of the authors and not necessarily those of the NHS, the National Institute for Health Research, or the Department of Health and Social Care. The study sponsor and funders had no role in study design; writing of the report; and the decision to submit the report for publication.

Data statement

Data sharing requests can be considered via contact with the corresponding author.

For peer review only

Competing interests statement

None declared.

References

1. Court-Brown CM, Caesar B. Epidemiology of adult fractures: a review. *Injury* 2006;37(8):691-97. doi: 10.1016/j.injury.2006.04.130
2. Curtis EM, van der Velde R, Moon RJ, et al. Epidemiology of Fractures in the United Kingdom 1988-2012: Variation with age, sex, geography, ethnicity and socioeconomic status. *Bone* 2016;87:19-26. doi: 10.1016/j.bone.2016.03.006
3. Kannus P, Palvanen M, Niemi S, et al. Stabilizing incidence of low-trauma ankle fractures in elderly people Finnish statistics in 1970-2006 and prediction for the future. *Bone* 2008;43(2):340-2. doi: 10.1016/j.bone.2008.04.015
4. National Institute for Clinical and Care Excellence. Osteoporosis: assessing the risk of fragility fracture Clinical guideline [CG146]2017.
5. Keene DJ, Mistry D, Nam J, et al. The Ankle Injury Management (AIM) Trial: A pragmatic, multi-centre, equivalence randomised controlled trial and economic evaluation comparing close contact casting with open surgical reduction and internal fixation in the treatment of unstable ankle fractures in patients over 60 years. *Health Technol Assess* 2016;20(75):1-158.
6. Willett K, Keene DJ, Mistry D, et al. Close Contact Casting vs Surgery for Initial Treatment of Unstable Ankle Fractures in Older Adults: A Randomized Clinical Trial. *Jama* 2016;316(14):1455-63. doi: 10.1001/jama.2016.14719
7. Lin CW, Moseley AM, Herbert RD, et al. Pain and dorsiflexion range of motion predict short- and medium-term activity limitation in people receiving physiotherapy intervention after ankle fracture: an observational study. *Aust J Physiother* 2009;55(1):31-7.
8. Psatha M, Wu Z, Gammie FM, et al. A longitudinal MRI study of muscle atrophy during lower leg immobilization following ankle fracture. *J Magn Reson Imaging* 2012;35(3):686-95. doi: 10.1002/jmri.22864
9. Wolf SI, Lessl E, Müller S, et al. Persistent diminished joint function following ankle fracture - a risk factor for posttraumatic osteoarthritis? [abstract]. *Gait Posture* 2013;38 (Suppl 1):S101-02. doi: http://dx.doi.org/10.1016/j.gaitpost.2013.07.207

For peer review only - http://bmjopen.bmj.com/site/about/guidelines.xhtml

10. Hancock MJ, Herbert RD, Stewart M. Prediction of outcome after ankle fracture. *J Orthop Sports Phys Ther* 2005;35(12):786-92.
11. Lin CW, Donkers NA, Refshauge KM, et al. Rehabilitation for ankle fractures in adults. *Cochrane Database Syst Rev* 2012;11:Cd005595. doi: 10.1002/14651858.CD005595.pub3
12. Moseley AM, Herbert RD, Nightingale EJ, et al. Passive stretching does not enhance outcomes in patients with plantarflexion contracture after cast immobilization for ankle fracture: a randomized controlled trial. *Arch Phys Med Rehabil* 2005;86(6):1118-26. doi: 10.1016/j.apmr.2004.11.017
13. Lin CW, Moseley AM, Haas M, et al. Manual therapy in addition to physiotherapy does not improve clinical or economic outcomes after ankle fracture. *J Rehabil Med* 2008;40(6):433-9. doi: 10.2340/16501977-0187
14. Nilsson GM, Jonsson K, Ekdahl CS, et al. Effects of a training program after surgically treated ankle fracture: a prospective randomised controlled trial. *BMC Musculoskelet Disord* 2009;10:118. doi: 10.1186/1471-2474-10-118
15. Moseley AM, Beckenkamp PR, Haas M, et al. Rehabilitation After Immobilization for Ankle Fracture: The EXACT Randomized Clinical Trial. *JAMA* 2015;314(13):1376-85. doi: 10.1001/jama.2015.12180
16. Dudek K, Druzbicki M, Przysada G, et al. Assessment of standing balance in patients after ankle fractures. *Acta Bioeng Biomech* 2014;16(4):59-65.
17. Keene DJ, Costa ML, Tutton E, et al. Progressive functional exercise versus best practice advice for adults aged 50 years or over after ankle fracture: protocol for a pilot randomised controlled trial in the UK - the Ankle Fracture Treatment: Enhancing Rehabilitation (AFTER) study. *BMJ Open* 2019;9(11):e030877. doi: 10.1136/bmjopen-2019-030877
18. Kanis JA, Cooper C, Rizzoli R, et al. European guidance for the diagnosis and management of osteoporosis in postmenopausal women. *Osteoporosis international : a journal established as result of cooperation between the European Foundation for Osteoporosis and the National Osteoporosis Foundation of the USA* 2019;30(1):3-44. doi: 10.1007/s00198-018-4704-5
19. Byrne C, Faure C, Keene DJ, et al. Ageing, muscle power, and physical function: A systematic review and implications for pragmatic training interventions. *Sports Med* 2016;In Press
20. National Institute for Clinical and Care Excellence. Falls in older people: Assessing risk and prevention. Manchester: NICE 2013.
21. Kraemer W, Adams K, Cafarelli E, et al. American College of Sports Medicine position stand. Progression models in resistance training for healthy adults. *Med Sci Sports Exerc* 2002;34(2):364-80.
22. Michie S, Rumsey N, Fussell A, et al. Improving health: Changing behaviour.: NHS health trainer handbook, 2008.
23. Thabane L, Ma J, Chu R, et al. A tutorial on pilot studies: The what, why and how. *BMC Med Res Methodol* 2010;10:1. doi: 10.1186/1471-2288-10-1
24. Olerud C, Molander H. A scoring scale for symptom evaluation after ankle fracture. *Arch Orthop Trauma Surg* 1984;103(3):190-4.
25. Binkley JM, Stratford PW, Lott SA, et al. The Lower Extremity Functional Scale (LEFS): Scale Development, Measurement Properties, and Clinical Application. *Phys Ther* 1999;79(4):371-83. doi: 10.1093/ptj/79.4.371
26. Brooks R. EuroQol: the current state of play. *Health Policy* 1996;37(1):53-72.

For peer review only

27. van Hout B, Janssen MF, Feng YS, et al. Interim scoring for the EQ-5D-5L: mapping the EQ-5D-5L to EQ-5D-3L value sets. *Value Health* 2012;15(5):708-15. doi: 10.1016/j.jval.2012.02.008
28. Kempen GIJM, Yardley L, Van Haastregt JCM, et al. The Short FES-I: a shortened version of the falls efficacy scale-international to assess fear of falling. *Age and Ageing* 2008;37(1):45-50. doi: 10.1093/ageing/afm157
29. Resnick B, Jenkins LS. Testing the Reliability and Validity of the Self-Efficacy for Exercise Scale. *Nurs Res* 2000;49(3):154-59.
30. Bennell KL, Talbot RC, Wajswelner H, et al. Intra-rater and inter-rater reliability of a weight-bearing lunge measure of ankle dorsiflexion. *Aust J Physiother* 1998;44(3):175-80.
31. Wang C-Y, Olson SL, Protas EJ. Test-retest strength reliability: hand-held dynamometry in community-dwelling elderly fallers. *Arch Phys Med Rehabil* 2002;83(6):811-15.
32. Guralnik JM, Simonsick EM, Ferrucci L, et al. A short physical performance battery assessing lower extremity function: Association with self-reported disability and prediction of mortality and nursing home admission. *J Gerontol* 1994;49(2):M85-M94. doi: 10.1093/geronj/49.2.M85
33. Schulz KF, Altman DG, Moher D. CONSORT 2010 statement: updated guidelines for reporting parallel group randomised trials. *BMJ* 2010;340:c332. doi: 10.1136/bmj.c332
34. Eldridge SM, Chan CL, Campbell MJ, et al. CONSORT 2010 statement: extension to randomised pilot and feasibility trials. *BMJ* 2016;355:i5239. doi: 10.1136/bmj.i5239
35. Hoffmann TC, Glasziou PP, Boutron I, et al. Better reporting of interventions: template for intervention description and replication (TIDieR) checklist and guide. *Bmj* 2014;348:g1687. doi: 10.1136/bmj.g1687
36. Morgan B, Hejdenberg J, Hinrichs-Krapels S, et al. Do feasibility studies contribute to, or avoid, waste in research? *PLoS One* 2018;13(4):e0195951. doi: 10.1371/journal.pone.0195951

For peer review only

For peer review only - http://bmjopen.bmj.com/site/about/guidelines.xhtml

TABLE 1: Intervention descriptions based on TIDieR guidance.

TIDieR Items[35]	Description	
**Brief Name**	AFTER (Ankle Fracture Treatment: Enhancing Rehabilitation)	
**Why**	Physiotherapy-led exercise and advice are commonly used to supervise rehabilitation after ankle fracture but evidence is lacking in terms of its benefit over advice on self-management.	
	**Progressive exercise**	**Best practice advice**
**What**	Home exercise and advice programme overseen by physiotherapist over≤6 sessions within 16 weeks.	Home exercise and advice programme initiated during a single face-to-face session with a physiotherapist, and then performed unsupervised by participant at home.
**Materials: Participants**	Participant Information Booklet and exercise instruction sheets with photos.<br>Action planner and exercise diary<br>Resistance bands (if applicable)	Participant Information Booklet incorporating exercise instructions with photos.
**Materials: Physiotherapists**	**Therapist training materials:** training session pack detailing study and intervention procedures and a quick reference guide detailing all aspects of the progressive exercise intervention.<br>**Training:** one day (2 to 3 hours) face-to-face training delivered by AFTER trial research physiotherapist. Training pack detailing all aspects of the trial and the progressive exercise intervention.	**Therapist training materials:** training session pack detailing study and intervention procedures.<br>**Training:** at least half a day. Training pack detailing all aspects of the trial and the best practice advice intervention.
**Procedures**	**Initial appointment**<br>Assess participant as per normal physiotherapy practice.<br>Issue folder containing the Progressive Exercise Participant Information Booklet.<br>Agree level of exercise that is most appropriate for the participant initially.<br>Advice should address barriers to exercise identified during assessment.<br>Provide education regarding pain during and after exercise.<br>Help participant to complete exercise documentation (exercise diary and action planner).<br>Make follow-up appointment(s).<br>Complete Treatment Log.<br><br>**Appointments 2-6**<br>Reassess as per normal physiotherapy practice.<br>Assist participant to progress/regress exercises.<br>Reassure the participant, reinforce key messages from the advice and education.<br>Review home exercise programme using the exercise diary.<br>Discuss return to functional activities.<br>Review action planner.	**Single face-to-face appointment**<br>Assess participant as per normal physiotherapy practice<br>Issue Best Practice Advice Participant Information Booklet.<br>Explain exercise difficulty level to start on based on assessment.<br>Educate participants how to progress and regress their exercises.<br>Advice should address barriers to exercise identified during assessment.<br>Provide education regarding pain during and after exercise.<br>Discharge participant with advice/encouragement to continue with the self-management exercise programme for at least 16 weeks.<br>Complete Treatment Log.<br><br>**Up to two further advice sessions (optional for participants having difficulties with self-management or the exercises)**<br><br>Physiotherapist's role was to re-assess and re-enforce self-management advice.

For peer review only - http://bmjopen.bmj.com/site/about/guidelines.xhtml

	Complete Treatment Log (after every session).	
**Who provides**	Physiotherapists already working in NHS musculoskeletal services who have attended the progressive exercise intervention training.<br>AFTER did not exclude physiotherapists based on number of years qualified or experience.	Physiotherapists already working in NHS musculoskeletal services who have attended the best practice advice intervention training.<br>AFTER did not exclude physiotherapists based on number of years qualified or experience.
**How**	Participants receive up to 6 sessions with a physiotherapist over 16 weeks.<br>The initial session 20 to 60 minutes for assessment. It is then followed by up to five sessions of up to 30 minutes each.	Participants receive a single face-to-face session with a physiotherapist lasting 20 to 60 minutes.<br>Up to two further advice sessions were optional for participants that having difficulties with self-management or the exercises.
**Where**	Physiotherapy sessions were in outpatient clinics based in the UK NHS.<br>Exercise programme is performed by the participant at home.	Same as progressive exercise intervention.
**When and how much**	Initial Appointment was as soon as possible after splint/cast removal, as per local appointment availability.<br>Up to 5 follow-up sessions arranged within 16 weeks.<br>Could be less than 6 sessions if participant has met rehabilitation goals and was self-managing condition.	Same as progressive exercise, but with option of one to two additional follow-up sessions if difficulties with self-management or the exercises.<br>Volume of exercise detailed in the manuscript.
**Tailoring**	**Education and advice**<br>Focus of education and advice were individualised based on assessment.<br>**Exercises**<br>Selection, manipulation of sets, repetitions and/or load is a joint decision-making process.<br>Range of motion and position could be modified to accommodate the patient's comfort and preferences.	**Education and advice**<br>Focus of education and advice are individualised based on assessment.<br>**Exercises**<br>The range of motion through which an exercise is performed, and the load and volume, could be increased or decreased.
**Modifications**	The optional follow-up sessions for best practice advice were initially set as face to face or telephone. Later in recruitment this was altered to telephone only.	
**Intervention Fidelity**		
**How well: <u>Training</u>**	All aspects of training delivery, content, structure, duration and therapists' confidence to implement the intervention were evaluated using post training feedback forms completed anonymously.	
**How well: <u>Physiotherapists</u>**	Intervention fidelity was monitored centrally via treatment logs, and during site visits.	
**How well: <u>Participants</u>**	**Exercise adherence**<br>Physiotherapists review the exercise diary at each subsequent session.<br>Participants asked to report exercise frequency in postal follow-up questionnaires at three and six months.	**Exercise adherence**<br>Participants asked to report exercise frequency in postal follow-up questionnaires at three and six months.
**How well: <u>Reporting</u>**	Intervention fidelity is reported in detail in this manuscript.	

For peer review only - http://bmjopen.bmj.com/site/about/guidelines.xhtml

TABLE 2: Baseline characteristics

	Best Practice Advice	Progressive Exercise	Total
Age (mean(SD))	63.3 (7.2)	63.7 (10.4)	63.5 (8.9)
Sex (n (%))			
Female	22 (73.3%)	20 (64.5%)	42 (68.9%)
Male	8 (26.7%)	11 (35.5%)	19 (31.1%)
BMI [1]	27.9 (4.8)	29.0 (6.8)	28.5 (5.9)
Ethnicity (n (%))			
Black or black British	1 (3.3%)	2 (6.5%)	3 (4.9%)
Mixed	1 (3.3%)	1 (3.2%)	2 (3.3%)
Other	1 (3.3%)	0 (0%)	1 (1.6%)
White British	27 (90%)	26 (83.9%)	53 (86.9%)
White other	0 (0%)	2 (6.5%)	2 (3.3%)
Education (n (%))			
Higher professional or university education	17 (56.7%)	14 (45.2%)	31 (50.8%)
None or Primary education	3 (10%)	0 (0%)	3 (4.9%)
Secondary education	10 (33.3%)	17 (54.8%)	27 (44.3%)
Smoking status (n (%))			
Current smoker	3 (10%)	4 (12.9%)	7 (11.5%)
Former smoker	10 (33.3%)	15 (48.4%)	25 (41%)
Never smoked	17 (56.7%)	12 (38.7%)	29 (47.5%)
Number of cigarettes per day (mean (SD))	9.3 (6.9)	17.5 (17.9)	14.2 (14.9)
Alcohol units per week (mean (SD))	11.1 (13.4)	5.5 (9.1)	8.2 (11.6)
Employment status (n (%))			
Employed	11 (36.7%)	14 (45.2%)	25 (41%)
Other	1 (3.3%)	0 (0%)	1 (1.6%)
Permanently sick or disabled	0 (0%)	1 (3.2%)	1 (1.6%)
Retired	14 (46.7%)	14 (45.2%)	28 (45.9%)
Semi-retired	4 (13.3%)	1 (3.2%)	5 (8.2%)
Unemployed	0 (0%)	1 (3.2%)	1 (1.6%)
Time off work (due to injury) (n (%))			
Not in paid work	13 (43.3%)	15 (48.4%)	28 (45.9%)
No	4 (13.3%)	3 (9.7%)	7 (11.5%)
Yes	12 (40%)	12 (38.7%)	24 (39.3%)
Work days missed (mean (SD))	16.1 (28.4)	19.3 (19.1)	17.9 (23.2)
Accommodation type (n (%))			
House/flat rented from housing association/local authority	3 (10%)	3 (9.7%)	6 (9.8%)
Other	2 (6.7%)	0 (0%)	2 (3.3%)
Owner occupied house/flat	21 (70%)	21 (67.7%)	42 (68.9%)
Privately rented house/flat	3 (10%)	7 (22.6%)	10 (16.4%)
Sheltered housing/warden control	1 (3.3%)	0 (0%)	1 (1.6%)
Unpaid carer (n (%))			
Yes	5 (16.7%)	9 (29.0%)	14 (23.0%)
No	25 (83.3%)	22 (71.0%)	47 (77.0%)
Resident unpaid carer (n (%))			
Yes	4 (13.3%)	5 (16.1%)	9 (14.8%)
No	1 (3.3%)	4 (12.9%)	5 (8.2%)
No unpaid carer	25 (83.3%)	22 (71.0%)	47 (77.0%)
Paid carer (n (%))			
Yes	2 (6.7%)	2 (6.5%)	4 (6.6%)
No	28 (93.3%)	28 (90.3%)	56 (91.8%)
Unknown/Missing	0 (0%)	1 (3.2%)	1 (1.6%)
Resident paid carer (n (%))			
Yes	1 (3.3%)	2(6.5%)	3 (4.9%)
No	1 (3.3%)	0 (0%)	1 (1.6%)
No paid carer	28 (93.3%)	28 (90.3%)	56 (91.8%)
Unknown/Missing	0 (0%)	1 (3.2%)	1 (1.6%)
Treatment type (n (%))			
Conservative (non-surgical) management	15 (50.0%)	13 (41.9%)	28 (45.9%)

For peer review only - http://bmjopen.bmj.com/site/about/guidelines.xhtml

	Best Practice Advice	Progressive Exercise	Total
Trad. Cast non-removable	3 (10.0%)	0 (0%)	3 (4.9%)
Trad. Case removable	1 (3.3%)	3 (9.7%)	4 (6.6%)
Close contact casting	5 (16.7%)	4 (12.9%)	9 (14.8%)
Walking boot	6 (20.0%)	6 (19.4%)	12 (19.7%)
Unknown/Missing	3 (10.0%)	3 (9.7%)	6 (9.8%)
Surgical management	12 (40.0%)	15 (48.4%)	27 (44.3%)
Clinical Outcomes (mean (SD))			
OMAS[2]	31.38 (17.97)	21.50 (15.71)	26.36 (17.44)
LEFS[3]	25.07 (13.39)	17.52 (11.68)	21.10 (12.98)
FES-I[4]	14.75 (5.18)	17.00 (6.16)	15.85 (5.74)
EQ-5D Utility[5]	0.48 (0.29)	0.25 (0.30)	0.36 (0.31)
EQ-5D VAS[6]	69.52 (19.11)	68.63 (20.04)	69.07 (19.42)
Pain at rest VAS[7]	26.6 (28.2)	32.1 (27.5)	29.4 (27.7)
Pain walking VAS[7]	37.8 (30.0)	55.2 (27.0)	46.2 (29.6)
Falls (last 3 months)	14.8 (5.2)	17.0 (6.2)	15.9 (5.7)
Falls that resulted in a broken bone (last 3 months) (n (%))			
No	12 (40%)	10 (32.3%)	22 (36.1%)
Yes[1]	2 (6.7%)	3 (9.7%)	5[1] (8.2%)
Unknown/Missing	16 (53.3%)	18 (58.1%)	34 (55.7%)
Aids pre-injury (n (%))			
Frame/rollator	8 (26.7%)	1 (3.2%)	2 (3.3%)
None	5 (16.7%)	27 (87.1%)	51 (83.6%)
One crutch	3 (10%)	1 (3.2%)	1 (1.6%)
One stick	3 (10%)	0 (0%)	3 (4.9%)
Two crutches	8 (26.7%)	1 (3.2%)	1 (1.6%)
Two sticks	1 (3.3%)	1 (3.2%)	1 (1.6%)
Unknown/Missing	2 (6.7%)	0 (0%)	2 (3.3%)
Aids since injury (n (%))			
Frame/rollator	8 (26.7%)	13 (41.9%)	21 (34.4%)
None	5 (16.7%)	1 (3.2%)	6 (9.8%)
One crutch	3 (10%)	1 (3.2%)	4 (6.6%)
One stick	3 (10%)		3 (4.9%)
Two crutches	8 (26.7%)	11 (35.5%)	19 (31.1%)
Two sticks	1 (3.3%)	2 (6.5%)	3 (4.9%)
Unknown/Missing	2 (6.7%)	3 (9.7%)	5 (8.2%)
Walking distance pre-injury (n (%))			
About the house	1 (3.3%)	2 (6.5%)	3 (4.9%)
Less than half a mile	3 (10%)	4 (12.9%)	7 (11.5%)
More than half a mile	24 (80%)	25 (80.6%)	49 (80.3%)
Bedbound	0 (0%)	0 (0%)	0 (0%)
Unknown/Missing	2 (6.7%)	0 (0%)	2 (3.3%)
Medication Use (n (%))			
Yes	21 (70%)	27 (87.1%)	48 (78.7%)
No	9 (30%)	4 (12.9%)	13 (21.3%)

1 BMI calculated as height divided by weight squared (m/kg^2)
2 OMAS: Olerud and Molander Ankle Score (0-100, higher scores better)
3 LEFS: Lower Extremity Functional Scale (0-100, higher scores better)
4 FES-I: Falls Efficacy Scale-International (short version): scores range from 7 (no concern about falling) to a maximum of 28 (severe concern about falling)
5 EQ-5D-5L utility, -0.594, indicating the worst possible health state, to 1.0 and is anchored at 0 [death] and 1.0 full health]
6 EQ-VAS gives self-rated health on a scale where the endpoints are labelled from 'worst imaginable health state' (0) to 'best imaginable health state' (100)
7 VAS: visual analogue scale, 0-100 scale, higher scores worse

For peer review only

For peer review only - http://bmjopen.bmj.com/site/about/guidelines.xhtml

TABLE 3: Compliance with the allocated treatment by trial arm

	Best Practice Advice	Progressive Exercise
**Totals**	**N = 30**	**N = 31**
Completed exercise treatment[1], n (%)	28 (93.3%)	18 (58.1%)
Partial exercise completion[2], n (%)	28 (93.3%)	28 (90.3%)
Received no treatment, n (%)	2 (6.7%)	3 (7.1%)
DNA/unable to contact	0 (0%)	1 (3.2%)
Withdrawal/declined	0 (0%)	0 (0%)
Too difficult to attend appointments	0 (0%)	1 (3.2%)
Unknown	2 (6.7%)	1 (3.2%)
**Median number of sessions (IQR)**	**1 (1, 2)**	**5 (3, 6)**
1 Sessions, n	20	3
2 Sessions, n	5	2
3 Sessions, n	-	3
4 Sessions, n	2	5
5 Sessions, n	1	7
6 Sessions, n	-	7
7 Sessions, n	-	1
Participants receiving additional sessions [3], n (%)	8 (26.7%)	1 (3.2%)
Before protocol change [4]	3	1
After protocol change [4]	5	0
Total number of additional contact sessions, n	13	1
Telephone	2	0
Face-to-face	11	1

[1] Best practice advice: attendance at ≥1 session; Progressive exercise: 6 sessions attended, or discharged by clinician as treatment completed (as marked on treatment log), or discharged by clinician following patient-initiated follow-up period with no further contact, or referred on for further investigation/treatment

[2] Defined as attendance at ≥1 session

[3] Additional sessions defined as >1 for BPA, and >6 for progressive exercise

[4] All the extra sessions for the BPA arm before the protocol change were face-to-face, of the five participants who got additional sessions after the protocol change, 2 of them were by telephone, and 3 were face-to-face

For peer review only - http://bmjopen.bmj.com/site/about/guidelines.xhtml

TABLE 4: Intervention delivery

Treatment components, N (%[1])	Best Practice Advice only<br>N (received BPA) = 28	Progressive exercise<br>N (received at least one session) = 28
Exercises prescribed	27 (96.4%)	23 (82.1%)
Information booklet provided	28 (100%)	24 (85.7%)
Session time in minutes (median, IQR)	45 (40, 60)	First session: 45 (45, 40)<br>Follow-up sessions: 30 (30, 45)

For peer review only

For peer review only - http://bmjopen.bmj.com/site/about/guidelines.xhtml

TABLE 5: Feasibility assessment

	Target	Outcome
Participation rate	A study participation rate of at least 25% of those eligible, to indicate acceptability and generalisability.	Sixty-one of 112 (54%) eligible participants participated in the trial, exceeding the feasibility criteria for participation of 25%.
Recruitment	At least 48 eligible participants across at least three sites agreed to participate over a maximum of 18 months.	61 participants were recruited in 11 months at 5 centres. There were more sites than originally planned so to interpret this feasibility criteria the recruitment rate per centre month was more appropriate. The recruitment feasibility criteria was based on 1.5 participants per site per month. 1.4 participants per centre month was achieved during the pilot trial.
Completion of study intervention sessions	At least 85% of participants completed study intervention sessions.	The intervention sessions were fully or partially delivery in 28/30 (93%) best practice advice and 28/31 (90%) progressive exercise participants.
Follow-up rate	At least 80% of participants attended study follow-up at 6 months.	Defined as the number who provided OMAS scores at 6 months, was 28/30 (93%) for the best practice advice group and 26/31 (84%) in the progressive exercise group, an overall follow-up rate of 89%.

For peer review only

TABLE 6: Summary statistics for each clinical outcome score

	Best Practice Advice			Progressive Exercise			Total		
	n	mean	SD	n	mean	SD	n	mean	SD
**OMAS[1]**									
Baseline	29	31.38	17.97	30	21.50	15.71	59	26.36	17.44
Month 3	28	65.54	20.29	26	62.50	23.59	54	64.07	21.79
Month 6	28	73.39	22.07	26	77.12	21.73	54	75.19	21.78
Change from baseline	27	43.70	14.58	25	55.00	24.87	52	49.13	20.79
**LEFS[2]**									
Baseline	28	25.07	13.39	31	17.52	11.68	59	21.10	12.98
Month 3	26	54.77	12.85	26	57.38	17.41	52	56.08	15.21
Month 6	25	65.36	11.45	27	61.52	20.05	52	63.37	16.44
Change from baseline	23	40.57	12.78	27	44.59	20.17	50	42.74	17.12
**FES-I[3]**									
Baseline	28	14.75	5.18	27	17.00	6.16	55	15.85	5.74
Month 3	25	10.04	3.22	26	9.65	3.72	51	9.84	3.45
Month 6	26	9.35	3.02	26	9.81	5.02	52	9.58	4.11
Change from baseline	26	-5.31	5.12	23	-8.30	5.34	49	-6.71	5.39
**EQ-5D Utility[4]**									
Baseline	29	0.48	0.29	30	0.25	0.30	59	0.36	0.31
Month 3	27	0.72	0.20	25	0.74	0.14	52	0.73	0.17
Month 6	28	0.77	0.15	28	0.81	0.27	56	0.79	0.22
Change from baseline	27	0.30	0.31	27	0.57	0.35	54	0.44	0.35
**EQ-5D VAS[5]**									
Baseline	29	69.52	19.11	30	68.63	20.04	59	69.07	19.42
Month 3	27	74.37	22.93	26	83.27	15.53	53	78.74	19.98
Month 6	28	82.07	17.85	28	81.36	18.21	56	81.71	17.87
**SPPB[6]**									
Month 6	22	10.27	1.91	25	10.24	1.59	47	10.26	1.73
Month 6[7]	27	8.48	4.41	28	9.36	3.60	55	8.93	4.01
**Range of Motion (RoM) (°)**									
dorsiflexion (uninjured)	24	10.8	5.4	26	12.5	8.7	50	11.7	7.3
dorsiflexion (injured)	24	9.2	5.1	26	7.6	9.0	50	8.4	7.3
dorsiflexion (difference)	24	1.7	3.3	26	4.9	8.1	50	3.3	6.4
**% RoM dorsal flexion[8]**		**85.2%**			**60.8%**			**71.8%**	
plantarflexion (uninjured)	24	47.8	17.0	26	43.5	14.7	50	45.6	15.9
plantarflexion (injured)	24	43.0	16.2	26	38.0	12.2	50	40.4	14.3
plantarflexion (difference)	24	4.8	10.1	26	5.5	7.0	50	5.2	8.5
**% RoM plantar flexion[8]**		**90.0%**			**87.4%**			**88.6%**	

For peer review only - http://bmjopen.bmj.com/site/about/guidelines.xhtml

	Best Practice Advice			Progressive Exercise			Total		
	n	mean	SD	n	mean	SD	n	mean	SD
inversion (uninjured)	24	33.5	10.1	26	31.5	10.8	50	32.5	10.4
inversion (injured)	24	26.0	10.7	26	26.3	11.7	50	26.2	11.1
inversion (difference)	24	7.5	8.0	26	5.2	8.7	50	6.3	8.4
**% RoM inversion[8]**		**77.6%**			**83.5%**			**80.6%**	
eversion (uninjured)	23	18.5	6.6	26	17.1	6.3	49	17.8	6.4
eversion (injured)	23	15.0	5.6	26	15.6	7.5	49	15.3	6.6
eversion (difference)	23	3.5	5.5	26	1.5	6.3	49	2.4	6.0
**% RoM eversion[8]**		**81.2%**			**91.2%**			**86.0%**	
**Strength[9] (Newtons)**									
Dorsiflexion strength	24	151.3	54.4	26	174.2	99.9	50	163.2	81.4
Plantarflexion strength	24	221.1	85.2	26	212.4	107.8	50	216.6	96.8
**Pain at rest VAS[10]**									
Baseline	29	26.6	28.2	31	32.1	27.5	60	29.4	27.7
Month 3	26	11.6	18.1	26	16.8	24.0	52	14.2	21.2
Month 6	25	6.5	13.6	28	8.2	22.7	53	7.4	18.8
**Pain walking VAS[10]**									
Baseline	28	37.8	30.0	26	55.2	27.0	54	46.2	29.6
Month 3	27	19.1	23.5	25	19.6	24.1	52	19.3	23.6
Month 6	26	12.2	19.6	27	10.7	22.8	53	11.4	21.1
**Self-Efficacy for Exercise Scale[11]**									
Month 3	18	61.7	27.2	20	71.0	22.0	48	66.6	24.7
Month 6	23	69.6	21.2	25	67.8	21.5	48	68.6	21.2

1 OMAS: Olerud and Molander Ankle Score (0-100, higher scores better)
2 LEFS: Lower Extremity Functional Scale (0-100, higher scores better)
3 FES-I: Falls Efficacy Scale-International (short version), scores range from 7 (no concern about falling) to a maximum of 28 (severe concern about falling).
4 EQ-5D-5L utility, -0.594, indicating the worst possible health state, to 1.0 and is anchored at 0 [death] and 1.0 full health].
5 EQ-VAS gives self-rated health on a scale where the endpoints are labelled from 'worst imaginable health state' (0) to 'best imaginable health state' (100) .
6 SPPB: Short Physical Performance Battery (scores range from 0 to 12, higher scores better).
7 Imputes 0 for those participants who completed form, but left questions blank – they were assumed to have not attempted
8 Calculated using the injured divided by the uninjured scores, multiplied by 100 to output as a percentage
9 The highest strength score (measured in Newtons N) out of 3 was used for each patient
10 VAS: visual analogue scale (0-100 scale, higher scores worse)
11 Self-efficacy exercise score. Overall scores range 0 to 90, with higher scores indicating greater confidence to exercise.

For peer review only

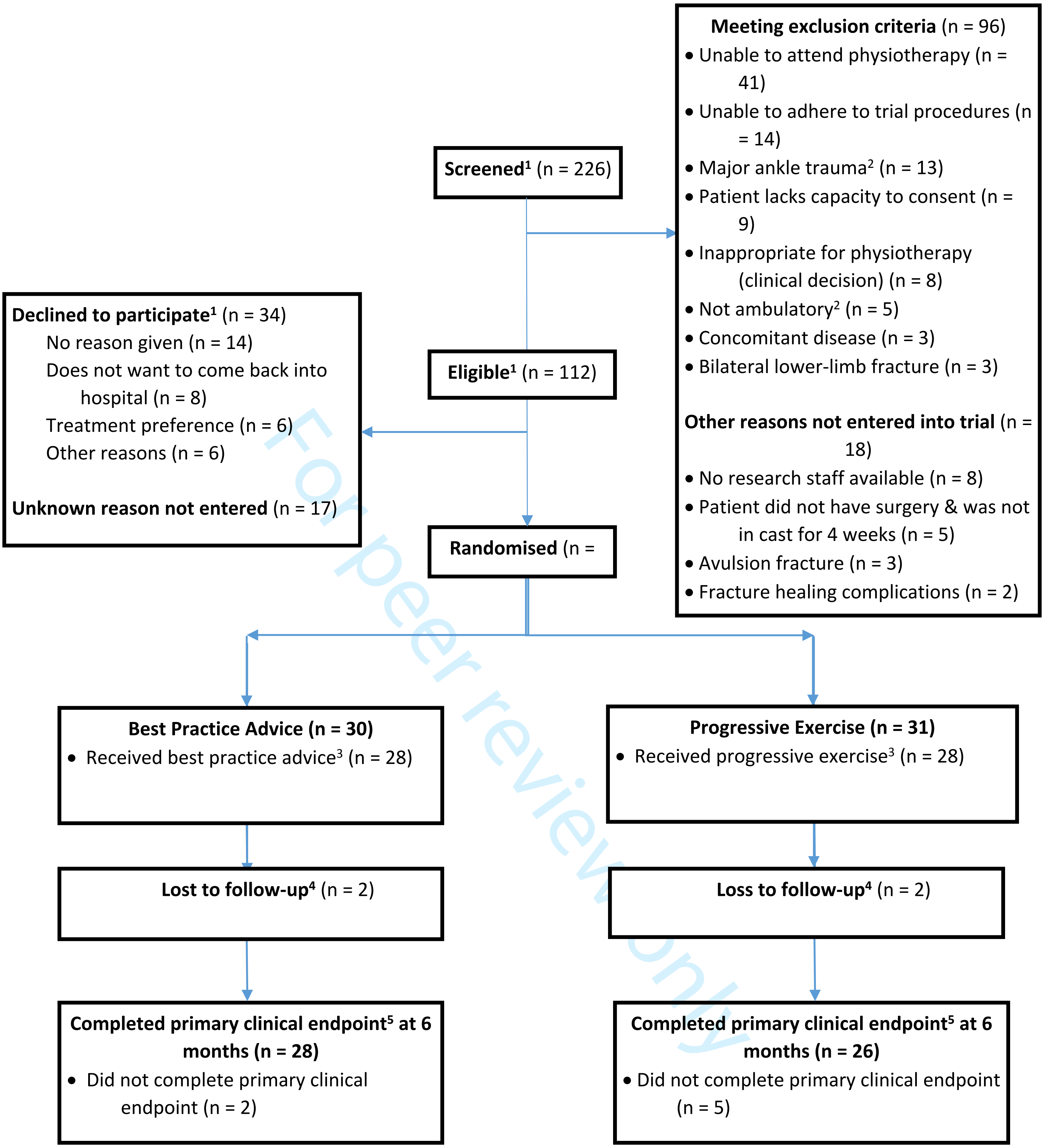

[1] One site has missing screening data for some of the randomised participants, so those who were randomised were added to the totals screened

[2] Major ankle trauma defined as: Open fracture wounds, external fixation, or substantial skin loss or grafts, that would limit ankle or lower leg exercise

[3] Compliance is defined as attending at least one session

*[4] Loss to follow-up is defined as **no response** at the 6 month follow-up*

[5] OMAS scores at 6 months

For peer review only

CONSORT 2010 checklist of information to include when reporting a pilot or feasibility trial*

Section/Topic	Item No	Checklist item	Reported on page No
**Title and abstract**			
	1a	Identification as a pilot or feasibility randomised trial in the title	1
	1b	Structured summary of pilot trial design, methods, results, and conclusions (for specific guidance see CONSORT abstract extension for pilot trials)	2
**Introduction**			
Background and objectives	2a	Scientific background and explanation of rationale for future definitive trial, and reasons for randomised pilot trial	4-5
	2b	Specific objectives or research questions for pilot trial	5
**Methods**			
Trial design	3a	Description of pilot trial design (such as parallel, factorial) including allocation ratio	5
	3b	Important changes to methods after pilot trial commencement (such as eligibility criteria), with reasons	7
Participants	4a	Eligibility criteria for participants	6
	4b	Settings and locations where the data were collected	5
	4c	How participants were identified and consented	5
Interventions	5	The interventions for each group with sufficient details to allow replication, including how and when they were actually administered	7-8, Table 1
Outcomes	6a	Completely defined prespecified assessments or measurements to address each pilot trial objective specified in 2b, including how and when they were assessed	9
	6b	Any changes to pilot trial assessments or measurements after the pilot trial commenced, with reasons	N/A
	6c	If applicable, prespecified criteria used to judge whether, or how, to proceed with future definitive trial	9
Sample size	7a	Rationale for numbers in the pilot trial	11
	7b	When applicable, explanation of any interim analyses and stopping guidelines	N/A
Randomisation:			
Sequence generation	8a	Method used to generate the random allocation sequence	6
	8b	Type of randomisation(s); details of any restriction (such as blocking and block size)	6
Allocation concealment mechanism	9	Mechanism used to implement the random allocation sequence (such as sequentially numbered containers), describing any steps taken to conceal the sequence until interventions were assigned	6

For peer review only

Implementation	10	Who generated the random allocation sequence, who enrolled participants, and who assigned participants to interventions	6
Blinding	11a	If done, who was blinded after assignment to interventions (for example, participants, care providers, those assessing outcomes) and how	6
	11b	If relevant, description of the similarity of interventions	7-8
Statistical methods	12	Methods used to address each pilot trial objective whether qualitative or quantitative	9
**Results**			
Participant flow (a diagram is strongly recommended)	13a	For each group, the numbers of participants who were approached and/or assessed for eligibility, randomly assigned, received intended treatment, and were assessed for each objective	12
	13b	For each group, losses and exclusions after randomisation, together with reasons	Figure 1
Recruitment	14a	Dates defining the periods of recruitment and follow-up	12
	14b	Why the pilot trial ended or was stopped	12
Baseline data	15	A table showing baseline demographic and clinical characteristics for each group	Table 2
Numbers analysed	16	For each objective, number of participants (denominator) included in each analysis. If relevant, these numbers should be by randomised group	12-14
Outcomes and estimation	17	For each objective, results including expressions of uncertainty (such as 95% confidence interval) for any estimates. If relevant, these results should be by randomised group	12-14
Ancillary analyses	18	Results of any other analyses performed that could be used to inform the future definitive trial	12-14
Harms	19	All important harms or unintended effects in each group (for specific guidance see CONSORT for harms)	14
	19a	If relevant, other important unintended consequences	N/A
**Discussion**			
Limitations	20	Pilot trial limitations, addressing sources of potential bias and remaining uncertainty about feasibility	16
Generalisability	21	Generalisability (applicability) of pilot trial methods and findings to future definitive trial and other studies	15-16
Interpretation	22	Interpretation consistent with pilot trial objectives and findings, balancing potential benefits and harms, and considering other relevant evidence	15-16
	22a	Implications for progression from pilot to future definitive trial, including any proposed amendments	16-17
**Other information**			
Registration	23	Registration number for pilot trial and name of trial registry	3
Protocol	24	Where the pilot trial protocol can be accessed, if available	Cited p.5
Funding	25	Sources of funding and other support (such as supply of drugs), role of funders	18
	26	Ethical approval or approval by research review committee, confirmed with reference number	3, 12

For peer review only

Citation: Eldridge SM, Chan CL, Campbell MJ, Bond CM, Hopewell S, Thabane L, et al. CONSORT 2010 statement: extension to randomised pilot and feasibility trials. BMJ. 2016;355.

*We strongly recommend reading this statement in conjunction with the CONSORT 2010, extension to randomised pilot and feasibility trials, Explanation and Elaboration for important clarifications on all the items. If relevant, we also recommend reading CONSORT extensions for cluster randomised trials, non-inferiority and equivalence trials, non-pharmacological treatments, herbal interventions, and pragmatic trials. Additional extensions are forthcoming: for those and for up to date references relevant to this checklist, see www.consort-statement.org.

For peer review only

BMJ Open

Progressive exercise versus best practice advice for adults aged 50 years or over after ankle fracture: the AFTER pilot randomised controlled trial

Journal:	*BMJ Open*
Manuscript ID	bmjopen-2021-059235.R1
Article Type:	Original research
Date Submitted by the Author:	18-Aug-2022

Complete List of Authors:	Keene, David; University of Oxford, Oxford Trauma and Emergency Care, Nuffield Department of Orthopaedics, Rheumatology and Musculoskeletal Sciences<br>Costa, Matthew; University of Oxford, Nuffield Department of Orthopaedics, Rheumatology, and Musculoskeletal Sciences<br>Peckham, Nicholas; University of Oxford<br>Tutton, Elizabeth; University of Oxford, NDORMS<br>Barber, Vicki; University of Oxford Nuffield Department of Orthopaedics Rheumatology and Musculoskeletal Sciences, Oxford Clinical Trials Unit<br>Dutton, Susan; University of Oxford, CSM<br>Hopewell, Sally; University of Oxford, Nuffield Department of Orthopaedics, Rheumatology and Musculoskeletal Sciences<br>Redmond, Anthony; University of Leeds, Leeds Institute of Rheumatology and Musculoskeletal Medicine<br>Willett, Keith; University of Oxford, Orthopaedic Trauma Surgery<br>Lamb, Sarah; University of Exeter, College of Medicine and Health; University of Oxford, NDORMS<br>trial collabororators, AFTER ; trial collabororators

**Primary Subject Heading**:	Rehabilitation medicine
Secondary Subject Heading:	Surgery, Sports and exercise medicine, Geriatric medicine
Keywords:	Foot & ankle < ORTHOPAEDIC & TRAUMA SURGERY, Rehabilitation medicine < INTERNAL MEDICINE, Clinical trials < THERAPEUTICS

SCHOLARONE™
Manuscripts

BMJ

I, the Submitting Author has the right to grant and does grant on behalf of all authors of the Work (as defined in the below author licence), an exclusive licence and/or a non-exclusive licence for contributions from authors who are: i) UK Crown employees; ii) where BMJ has agreed a CC-BY licence shall apply, and/or iii) in accordance with the terms applicable for US Federal Government officers or employees acting as part of their official duties; on a worldwide, perpetual, irrevocable, royalty-free basis to BMJ Publishing Group Ltd ("BMJ") its licensees and where the relevant Journal is co-owned by BMJ to the co-owners of the Journal, to publish the Work in this journal and any other BMJ products and to exploit all rights, as set out in our licence.

The Submitting Author accepts and understands that any supply made under these terms is made by BMJ to the Submitting Author unless you are acting as an employee on behalf of your employer or a postgraduate student of an affiliated institution which is paying any applicable article publishing charge ("APC") for Open Access articles. Where the Submitting Author wishes to make the Work available on an Open Access basis (and intends to pay the relevant APC), the terms of reuse of such Open Access shall be governed by a Creative Commons licence – details of these licences and which Creative Commons licence will apply to this Work are set out in our licence referred to above.

Other than as permitted in any relevant BMJ Author's Self Archiving Policies, I confirm this Work has not been accepted for publication elsewhere, is not being considered for publication elsewhere and does not duplicate material already published. I confirm all authors consent to publication of this Work and authorise the granting of this licence.

For peer review only

Progressive exercise versus best practice advice for adults aged 50 years or over after ankle fracture: the AFTER pilot randomised controlled trial

David J. Keene[1*]
Matthew L. Costa[1]
Nicholas Peckham[2]
Elizabeth Tutton[1,3]
Vicki S. Barber[2]
Susan J. Dutton[2]
Sally Hopewell[2]
Anthony C. Redmond[4]
Keith Willett[1,2]
Sarah E. Lamb[5]
on behalf of the AFTER pilot trial collaborators

[1] Oxford Trauma and Emergency Care, Nuffield Department of Orthopaedics, Rheumatology and Musculoskeletal Sciences, University of Oxford, Oxford, UK
[2] Oxford Clinical Trials Research Unit (OCTRU), University of Oxford, Oxford, UK
[3] Oxford University Hospitals NHS Foundation Trust, Oxford, UK
[4] Leeds Institute of Rheumatic and Musculoskeletal Medicine, University of Leeds, Leeds, UK
[5] College of Medicine and Health, University of Exeter, Exeter. UK

Word count:

*corresponding author
Dr David J. Keene
Kadoorie Centre, Level 3, John Radcliffe Hospital, OX3 9DU
david.keene@ndorms.ox.ac.uk

For peer review only

ABSTRACT

Objective

The aim of the Ankle Fracture Treatment: Enhancing Rehabilitation (AFTER) study, a multicentre external pilot parallel-group randomised controlled trial (RCT), was to assess feasibility of a definitive trial comparing rehabilitation approaches after ankle fracture.

Setting

Five UK National Health Service hospitals

Participants

Participants were aged 50 years and over with an ankle fracture requiring immobilisation for at least 4 weeks.

Interventions

Participants were allocated 1:1 via a central web-based randomisation system to: i) best-practice advice (one session of physiotherapy, up to two optional additional advice sessions) or ii) progressive exercise (up to six sessions of physiotherapy).

Primary outcome measures:

Feasibility: i) participation rate ii) intervention adherence iii) retention.

Results

Sixty-one of 112 (54%) eligible participants participated, exceeding progression criteria for participation of 25%. Recruitment progression criteria was 1.5 participants per site per month, 1.4 was observed. At least one intervention session was delivered for 28/30 (93%) of best practice advice and 28/31 (90%) of progressive exercise participants, exceeding the 85% progression criteria. For those providing follow-up data, the proportion of participants reporting performance of home exercises in the best practice advice and progressive exercise groups at three months was 20/23 (87%) and 21/25 (84%) respectively. Mean time from injury to starting physiotherapy was 74.1 days (95%CI 53.9 to 94.1 days) for best practice advice and 72.7 days (95%CI 54.7 to 88.9) for progressive exercise. Follow-up rate (6 month Olerud Molander Ankle Score) was 28/30 (93%) for the best practice advice group and 26/31 (84%) in the progressive exercise group, an overall follow-up rate of 89%.

For peer review only - http://bmjopen.bmj.com/site/about/guidelines.xhtml

For peer review only

Conclusions

This pilot RCT demonstrated a definitive trial would be feasible. The main issues to address for a definitive trial are intervention modifications to enable earlier provision of rehabilitation and ensuring similar rates of follow-up in each group.

Trial registration: ISRCTN16612336

Keywords

Ankle Fractures, Clinical Trial, Geriatrics, Exercise Therapy

Strengths and limitations of this study

- Our pilot RCT demonstrated that a definitive trial comparing a best practice advice versus a supervised progressive exercise intervention for adults aged 50 years and over after ankle fracture is feasible.
- All feasibility success criteria were met, apart from a minor difference in the recruitment rate per site-month that would need to be factored into planning a definitive trial.
- Follow-up rates differed between the groups, ensuring similar rates in a definitive trial is critical.
- A limitation of the study, common to all pilot trials, is that with the necessarily limited number of centres, there remains some uncertainty regarding the feasibility of a trial across a much larger number of centres.

For peer review only

INTRODUCTION

Ankle fractures account for 9% of all fractures managed in secondary care.[1] In the UK, incidence of these fractures is highest in people aged 50 years and over, peaking at 16 per 10,000 person-years in women aged 60 to 70.[2] As the population ages, a three-fold increase in these fractures is projected over the next two decades.[3] The mechanism of injury for people aged over 50 is commonly a fall from standing height, which are defined as fragility fractures.[4]

Treatments for ankle fractures range from conservative plaster casts or boots to surgical fixation. The Ankle Injury Management (AIM) trial found that regardless of the initial fracture management, at 6 months reduced ankle function and walking abnormalities are substantial.[5 6] Participants reported an average 30% loss of pre-injury ankle function. Function is poor due to pain and reduced joint motion,[7] lower limb muscle strength deficits,[8] gait abnormalities,[9] and resultant mobility limitations.[7 10]

Weight bearing and ankle movement restrictions are usually removed by the orthopaedic team 6 weeks after injury. A Cochrane review[11] of ankle fracture rehabilitation concluded that there was insufficient evidence to support traditional physiotherapy interventions targeting ankle joint and muscle impairments, such as stretching,[12] manual therapy,[13] and exercise.[14] Updating the Cochrane review searches in MEDLINE and EMBASE, identified another multicentre trial by Moseley and colleagues.[15] They found no differences in self-reported lower limb function or quality of life between supervised exercise and a one-off advice session for adults with ankle fractures treated surgically and conservatively. Physiotherapists delivered both interventions face-to-face and the mean age of participants was 42 years.

Physiotherapy interventions tested to date have not incorporated features of advice and exercise programmes for older adults used in other rehabilitation areas. Issues such as persistent poor balance in older adults require attention.[16] The Ankle Fracture Treatment: Enhancing Rehabilitation (AFTER) study was a pilot randomised controlled trial to assess

For peer review only - http://bmjopen.bmj.com/site/about/guidelines.xhtml

feasibility of a definitive trial comparing best-practice advice with a supervised progressive exercise programme for adults aged 50 years and over after ankle fracture.

Feasibility Objectives

The main objectives of the pilot trial were to:

- Assess patient engagement with the trial, measured by the participation rate of those eligible.
- Establish whether the interventions were acceptable to participants and therapists, measured by intervention adherence levels (and participant interviews and a therapist focus group – reported separately).
- Determine patient retention, measured by the proportion of patients providing outcome data at 6 months
- Assess the acceptability of measuring outcomes at 3 and 6 months post randomisation, measured by outcome measure data collection.

METHODS

Study design

A multicentre pilot parallel-group RCT. Participants were allocated to either: i) best-practice advice (one session of face-to-face advice delivered by a physiotherapist, with up to two further optional advice sessions as deemed required by the physiotherapist) or ii) progressive exercise (up to six sessions of individual face-to-face physiotherapy). The pilot trial protocol has been published.[17] An embedded qualitative study including patient participants and staff was also completed and will be reported separately.

Setting

Recruitment was from five NHS hospitals. Participants were identified while inpatients or when attending fracture clinics, where they were screened and given study information. Patients who meet the eligibility criteria and wanted to participate were approached for written informed consent.

For peer review only - http://bmjopen.bmj.com/site/about/guidelines.xhtml

Study participants

The target population was adults aged 50 years as bone density reduces at this age and there is an increased risk of fragility fractures.[18] There is also a bimodal age distribution for ankle fractures, with peak incidences being for young adult males, and highest for females aged 50 years and over.[2] Participants were attending NHS services to manage ankle fractures that either required definitive management with surgical, or non-surgical management of ankle immobilisation for at least 4 weeks.

Patients were excluded if they:

- were unable to adhere to trial procedures or complete questionnaires
- did not have capacity to consent to study participation
- were not ambulatory before the injury
- were considered inappropriate for referral to physiotherapy by the clinician
- could not attend outpatient physiotherapy at a participating centre
- had serious concomitant disease (such as terminal illness)
- had bilateral lower limb fractures
- had an ipsilateral concurrent Pilon fracture
- had open fracture wounds, external fixation, or substantial skin loss or grafts that would limit ankle or lower leg exercise

Randomisation

Participants were randomised 1:1 to the intervention groups using the centralised computer randomisation service provided by the Oxford Clinical Trials Research Unit. The site's research facilitator undertook randomisation directly themselves or contacted the study office over the telephone to access the system on their behalf. Randomisation was computer-generated and stratified by centre and initial fracture management (surgery or non-surgical) using variable block sizes (2 and 4).

For peer review only - http://bmjopen.bmj.com/site/about/guidelines.xhtml

Blinding

Physiotherapists delivering the intervention and study participants were told the treatment allocation. Physiotherapists or trained research associates independent of the clinical team collected the objective outcome measures at the 6-month follow-up time point.

Interventions

The best-practice advice or progressive exercise sessions were given when the participant could mobilise with unrestricted weight-bearing and do ankle exercises as guided by their surgeon or physiotherapist. We anticipated that this would usually be around 6-8 weeks post-injury. Both interventions were delivered face-to-face and one-to-one by a physiotherapist. The interventions are outlined in Table 1. In summary:

Best-practice advice

The best-practice advice intervention included a 20 to 60 minute session (depending on local service provision) of assessment, education, reassurance, and detailed self-management advice on ankle exercises, gait training, stair climbing, walking aid advice, and basic balance exercises. An advice booklet provided a summary of key information.

Up to two further advice sessions were optional for participants having difficulties with self-management or the exercises. The physiotherapist's role was to re-assess and re-enforce self-management advice. During the initial stage of recruitment these additional sessions involved a telephone call or an additional face-to-face contact. In the latter stage of recruitment (after 41 participants had been recruited), these sessions were to be offered as telephone consultations only. This change was introduced to assess whether a greater focus on the single face-to-face session was acceptable to trial participants and physiotherapists. Use of additional sessions were recorded and monitored.

Progressive exercise

A physiotherapist instructed participants and supervised their progressive exercise programme in up to six sessions over 16 weeks. This period aimed to allow sufficient time for neuromuscular adaptation to exercise.[19] The programme could end early if all

For peer review only - http://bmjopen.bmj.com/site/about/guidelines.xhtml

rehabilitation goals were achieved in under six sessions. The first session was 20-60 minutes and the rest were up to 30 minutes, consistent with the duration of physiotherapy sessions in the NHS. Physiotherapists provided assessment, advice, education about progressing recovery, gait training, walking aid instruction, and an information booklet.

The programme was highly structured but calibrated for each individual. All participants received a core set of functional lower limb resistance exercises in line with the evidence for improving muscular strength and power in older persons' rehabilitation.[20] The programme included supervised gait training to target major walking difficulties after ankle fracture. Balance exercises were included in the programme and introduced once the participant was able to weight bear sufficiently to perform these. Exercises were practised in the clinic but conducted at home with the aim of achieving an effective dose. Based on the participant's functional goals, exercises were progressed to make them task-specific, for example walking on uneven surfaces or slopes, climbing stairs, or jogging. Participants received an exercise planner and diary. Exercise progression was based on evidence-based guidelines[21] but individualised by progressing and regressing the volume and load in line with each participant's capabilities and preferences.

The progressive exercise intervention used simple health behaviour change techniques to optimise adherence to home exercise.[22] Participants were asked to identify their goals following usual physiotherapy practice and, with the treating therapist's help, write an action plan for where and when they would perform their home exercises and a contingency plan for managing difficulties. Therapists were trained to focus on helping participants identify barriers to exercise and becoming more physically active post-injury, and facilitating problem-solving. A high-quality information booklet was developed by the AFTER study team with Patient and Public Involvement representatives and provided to participants.

Training and monitoring of intervention delivery

Treating therapists were trained in a face-to-face session of up to four hours and provided with written materials on the theory and practical delivery of the interventions. The same therapists were trained in both interventions by the trial research physiotherapist (an

For peer review only - http://bmjopen.bmj.com/site/about/guidelines.xhtml

experienced clinician with specialist post-graduate musculoskeletal specialist training, who was also the chief investigator for the study). Intervention delivery was recorded in logs for each contact with participants to monitor intervention fidelity and enable ongoing feedback to manage contamination. Local site Principal Investigators were also responsible for monitoring intervention delivery.

Best practice advice delivery adherence was defined as attendance of at least one physiotherapy session. For progressive exercise delivery, adherence was defined as attending all six sessions, or if they were discharged by the physiotherapist as treatment completed (as marked on treatment log), or discharged by the physiotherapist following a patient-initiated follow-up period with no further contact, or if they were referred on for further investigations/treatment. Partial adherence for progressive exercise was attending at least one session but not meeting the outlined criteria for full adherence. Additional sessions were recorded for any best practice advice sessions after the main first sessions and for progressive exercise it as any sessions in addition to the six sessions. For the progressive exercise programme, treatment logs captured exercise prescription details so that progression of the programme over the sessions in terms of exercise type, volume or load could be assessed.

Concomitant care

Other aspects of health and social care continued as normal. Additional treatments, including contact with their GP or other health professionals, were recorded in participant follow-up questionnaires.

Outcome measures

Feasibility success criteria

The main aim of this pilot RCT was to determine the feasibility of a future definitive trial.[23] The main uncertainty was whether patients find it acceptable to be randomised to different types of rehabilitation provision. To determine the feasibility of a definitive RCT, the pre-specified success criteria[17] were:

- A study participation rate of at least 25% of those eligible

For peer review only - http://bmjopen.bmj.com/site/about/guidelines.xhtml

- At least 48 eligible patients across at least three sites agree to participate over a maximum of 18 months
- At least 85% of participants completed study intervention sessions
- At least 80% of participants attended study follow-up at 6 months, defined as the proportion of participants who provided OMAS scores at 6 months

Outcomes

Participants were followed up 3 months after randomisation with a postal questionnaire and 6 months after randomisation face-to-face at the hospital. They were offered telephone or postal follow-up if they were unable to attend the 6-month follow-up.

Patient-reported outcomes at 3 and 6 months were:

- ankle-related symptoms and function: Olerud and Molander Ankle Score (OMAS, 0-100, higher scores better)[24]
- lower-limb function limitations: Lower Extremity Functional Scale (0-100, higher scores better)[25]
- pain: visual analogue scale (VAS), 0-100 scale, higher scores worse
- health-related quality of life: EQ-5D-5L score.[26] The EQ-5D health status scale is mapped onto the EQ-5D-3L valuation set using the Crosswalk Index Value Calculator.[27] The scale from this set ranges from -0.594, indicating the worst possible health state, to 1.0 and is anchored at 0 [death] and 1.0 full health]. A respondent's EQ-VAS gives self-rated health on a scale where the endpoints are labelled from 'worst imaginable health state' (0) to 'best imaginable health state' (100).
- fear of falls: Falls Efficacy Scale-International (short version).[28] The overall scores range from 7 (no concern about falling) to a maximum of 28 (severe concern about falling).
- self-efficacy: self-efficacy exercise score.[29] Overall scores range 0 to 90, with higher scores indicating greater confidence to exercise.
- self-reported return to desired activities, including work, social life, and sport activities
- walking aid use and distances

For peer review only - http://bmjopen.bmj.com/site/about/guidelines.xhtml

- exercise adherence (self-reported exercise frequency)
- health resource use (including additional out of trial physiotherapy).

At 6-month follow-up, a blinded outcome assessor collected objective measures of ankle function and physical performance:

- ankle joint range: hand-held goniometry[30]
- muscle strength: hand-held dynamometry (Lafayette Manual Muscle Test System, Lafayette Instrument, IN, USA) of ankle dorsi/plantarflexion using a 'make' approach (working up to a maximal contraction over a maximum of 5 seconds and without pushing into pain and the assessor maintaining position of the device).[31] Participants were measured 3 times and had at least 10 seconds rest between attempts.
- mobility and balance: Short Physical Performance Battery (SPPB).[32] The test involves physical tests of balance, walking speed, and repeated rises from a chair.

Adverse events

Expected general side effects of any exercise, such as delayed-onset muscle soreness and temporary increases in pain of less than a week, were not recorded as adverse events. Pain increases of more than a week were recorded in patient-reported questionnaires. Any exacerbations of other medical conditions during exercise or exercise-related injurious falls could also be recorded in patient-reported questionnaires, or by the site investigators if they became aware of such an event.

Sample size

The main feasibility objective and therefore the basis of the sample size estimate was participant recruitment per centre. The target sample size was a minimum of 48 participants in at least three centres over a maximum of 18 months. The sample size was based on a target of recruiting 1.5 participants per month per site. An amendment to the protocol during recruitment enabled the recruitment to continue up to 60 participants so that more feasibility data could be collected after the changes to the best practice advice intervention outlined above.

For peer review only - http://bmjopen.bmj.com/site/about/guidelines.xhtml

Statistical analysis

Baseline characteristics and outcomes were reported using descriptive statistics using mean and standard deviation (or median and interquartile range if non-normally distributed), and minimum and maximum, for continuous variables and number and percentage of participants in each group for binary or categorical variables. Feasibility outcomes were reported as numbers and percentages and compared to the progression criteria targets. Outcome data were analysed on an intention-to-treat basis, with all participants analysed as per their treatment group allocation and differences in outcomes between treatment groups were reported with 95% confidence intervals. As the study was not powered for formal hypothesis testing between the treatment arms no p-values are provided.

Research ethics approval

The study was approved by the South Central – Hampshire B Research Ethics Committee (Ref: 18/SC/0281) on 2nd July 2018 and the Health Research Authority and was prospectively registered (ISRCTN16612336).

We used the Consolidated Standards of Reporting Trials (CONSORT) guidelines for pilot and feasibility trials[33 34] and the Template for Intervention Description and Replication (TIDieR) statement to report the study.[35]

Patient and Public Involvement

The intervention development and study materials were supported by a patient and public involvement group, who were also involved in interpreting the study findings and definitive trial protocol design.

RESULTS

Screening, recruitment and baseline characteristics

Patient enrolment began in October 2018 and was completed in August 2019 when the sample size was reached. Of the 226 patients screened, 112 were eligible, and 61 were recruited and randomised (see Figure 1). The main reason screened patients were not eligible was that they were unable to attend outpatient physiotherapy at a participating centre (41/114, 36%). Of the 34 patients who declined participation, the main reason

For peer review only - http://bmjopen.bmj.com/site/about/guidelines.xhtml

provided was that they did not want to return to the participating centre for the study due to practical difficulties such as transport and parking (8/34, 24%). The revised sample size target of 60 was exceeded as one participant had consented to participation and randomised before closure of the randomisation system.

Baseline characteristics are summarised in Table 2. Participants had a mean age of 63.5 (SD 8.9) years, and 42/61 (69%) were female. 41/61 (67%) had surgery and the remainder (20/61, 33%) had non-surgical treatment for their ankle fracture.

Feasibility outcomes

Patient engagement with the trial: The final recruitment rate of eligible participants was 54%. The overall recruitment rate per month per site was 1.4 participants.

Intervention adherence levels: The mean time from randomisation to having the first physiotherapy session was 39.2 days (95% CI 27.3 to 55.3) for best practice advice and 38.2 (95% CI 29.8 to 48.1) for progressive exercise. From injury to starting physiotherapy it was 74.1 days (95% CI 53.9 to 94.1 days) for best practice advice and 72.7 days (95% CI 54.7 to 88.9) for progressive exercise.

At least one intervention session was delivered in 28/30 (93%) best practice advice and 28/31 (90%) progressive exercise participants. 18/31 (58%) participants met the criteria for fully completing the progressive exercise intervention (see Table 3). Overall, the levels of fidelity in delivery of the intervention components were high, see Table 4. The median number of physiotherapy sessions for best practice advice was 1 (IQR 1,2) and for progressive exercise it was 5 (IQR 3, 6).

For the progressive exercise programme, there was evidence in treatment logs of exercise prescription progression (exercise type, volume or load) across sessions for 21/28 (75%) of participants. Exercises were not progressed for one participant, and none regressed over the sessions. It was not possible to assess for six participants due to insufficient exercise prescription data.

For peer review only - http://bmjopen.bmj.com/site/about/guidelines.xhtml

At 3 months post-randomisation, the proportion of participants performing home exercises out of those providing data in the best practice advice and progressive exercise groups was 20/23 (87%) and 21/25 (84%) respectively. At six months this reduced to 14/26 (54%) in the best practice advice group and 19/26 (73%) in the progressive exercise group.

Of the 28 participants allocated to best practice advice group that received at least the first session, overall, 13 (46%) had further contacts with their physiotherapist. Eight (29%) had further contacts with their physiotherapist prior to the protocol change (see Methods). Four participants that had an additional physiotherapy session (4/28, 14%) had clinical reasons for having a further face to face session (no more than two sessions provided) due to issues with pain and concerns cited regarding progress and discolouration of the ankle. The other four participants had issues with appointment timing and scheduling, two participants needed extra face-to-face sessions as they had their first sessions scheduled too early (before they were permitted to weight bear), another participant arrived too late for their appointment so had to come back to complete delivery, and one participant was initially seen in physiotherapy in error and had a usual care physiotherapy appointment, they had an extra session to receive the best practice advice session. Of the five participants who had additional contact with their physiotherapist after the protocol change, two were by telephone, and three were face-to-face. One participant in the progressive exercise group had a further contact in addition to the six physiotherapy sessions. Three participants in each group reported receiving sessions of physiotherapy outside the trial (see health resource use data in the supplementary appendix Table S1 and S2).

Patient retention and outcome measure data collection: The patient-reported outcomes and objective measures of ankle function and physical performance are reported in Table 5. Follow up rate for OMAS scores at 6 months was 28/30 (93%) for the best practice advice group and 26/31 (84%) in the progressive exercise group, an overall follow-up rate of 89%. Completion rates for the objective outcome assessments at 6 months were lower than for the OMAS (e.g., 47/61 [77%] for SPPB). Data on return to desired activities, including work, social life, and sport activities, walking aid use and distances, and health resource use are reported in the supplementary appendix (Table S1 to S4).

For peer review only - http://bmjopen.bmj.com/site/about/guidelines.xhtml

Adverse events: No serious adverse events were reported. Increases in pain of more than one week were reported by three participants in the best practice advice group and two in the progressive exercise group, all at the three-month timepoint. One participant in the progressive exercise group consulted a physician due to this symptom increase. No increases in pain were reported at 6 months. Five participants reported a worsening of a medical condition at three months (three in the best practice advice group and two in the progressive exercise group). Of these reports, 3/5 (60%) sought medical attention as a result. One participant in the best practice group reported a worsening of a medical condition at six months and saw a physician. Adverse events are reported in the supplementary appendix Table S5.

A summary of the pre-specified feasibility success criteria and the respective outcomes are shown in Table 6.

DISCUSSION

Our pilot RCT demonstrated that a definitive trial comparing a best practice advice versus a supervised progressive exercise intervention for adults aged 50 years and over after ankle fracture is feasible. All feasibility success criteria were met, apart from a minor difference in the recruitment rate per site-month that would need to be factored into planning a definitive trial. Additionally, ensuring similar rates of follow-up in each intervention group is critical.

Main areas of refinement for the definitive RCT indicated by the pilot related to the interventions. Most patients had to wait several weeks after removal of the cast/splint for their first physiotherapy session. It would therefore be ideal for the early rehabilitation advice to start at the point where the cast or splint are removed rather than having to wait for a separate outpatient physiotherapy appointment. The most common reason participants did not want to participate was the requirement to attend the hospital for appointments. With the now widespread uptake of videoconferencing to deliver physiotherapy in the UK, a future trial where this is available as an option could make the trial more inclusive.

For peer review only - http://bmjopen.bmj.com/site/about/guidelines.xhtml

For peer review only

In the AFTER pilot RCT we observed that in the best practice advice group about two in three participants had a single session of advice and did not use the optional additional sessions. Half of the additional sessions had clinical reasons, the remainder had issues with appointment timing and scheduling. The clinical reasons provided for the additional session seemed to relate to concerns regarding progress and symptoms that were concerning but anticipated after ankle fracture. The advice intervention could be improved with further information on symptoms and rate of progress to help address these early concerns. The majority of participants managed with a single session of advice without initiating the optional extra sessions. There was also minimal and balanced use of physiotherapy outside the trial over the six months (three participants in each intervention group, 6/61 [10%] in total). This contrasts to the previously largest trial of advice versus supervised rehabilitation, the EXACT trial that was based in Australia,[15] where 39/108 (36%) of the advice group and 15/106 (14%) of the physiotherapy supervised rehabilitation group had additional physiotherapy outside the trial, 54/214 (25%) in total.

In comparison to the outcome measure completion rates for patient-reported outcomes, there were lower rates of objective measures of ankle function and physical performance. Given the greater extent of missing outcome data and the extra clinical and participant burden, and the context of trying to reduce hospital visits since the start of the COVID-19 pandemic, use of remote follow-up with questionnaires only is an important option for the definitive RCT.

We asked participants to self-report adverse events as one of the interventions had a much greater level of contact with a health professional and it was anticipated that there would be few serious adverse events. However, the validity of the relatedness of adverse events to the participants rehabilitation is difficult to assess when dependant on self-report. The recruiting centres could also report adverse events, this aimed to identify the more serious treatment-related complications. The progressive exercise programme had a higher intensity than the best practice advice programme. The higher intensity appeared to be generally well tolerated as there were few adverse events, no serious adverse events, and those events reported were similar between the intervention groups.

For peer review only - http://bmjopen.bmj.com/site/about/guidelines.xhtml

A limitation of the study, common to all pilot trials is that with the necessarily limited number of centres, there remains some uncertainty regarding the feasibility of a trial across a much larger number of centres. We attempted to mitigate this issue by running the trial at a range of trauma units and major trauma centres. These centres had varied geographical settings, diverse populations, and a range of levels of experience in running multicentre trauma trials. Physiotherapists were trained in both interventions so there was a potential risk of contamination. Although treatment logs indicated good levels of intervention fidelity, more onsite observation and/or recording of interventions would have enabled a more robust assessment of fidelity. Training separate physiotherapists in each intervention would also be advisable where it is practical to do so to further reduce the risk of contamination.

The evidence from this pilot RCT demonstrated that with refinement, a definitive trial would be feasible. A definitive trial was designed based on the findings from the external pilot and has subsequently been funded (National Institute for Health and Care Research reference: NIHR201950). The definitive AFTER trial will assess the clinical effectiveness of physiotherapist-supervised rehabilitation versus self-directed rehabilitation for adults aged 50 years and over after ankle fracture. The interventions, study process and outcome assessments have all been refined for the full trial. The primary endpoint will be the OMAS at six months. The full AFTER id due to complete in July 2024.

For peer review only - http://bmjopen.bmj.com/site/about/guidelines.xhtml

Acknowledgements

The AFTER study collaborating Principal Investigators were:

Hannah Perkins (Oxford University Hospitals NHS Foundation Trust)

Carol McCrum (East Sussex Healthcare NHS Trust)

Jacky Jones (Guy's and St Thomas' NHS Foundation Trust)

Carey McClellan and Naomi Chalk (University Hospitals NHS Foundation Trust)

Fiona Cowell (Royal Liverpool and Broadgreen University Hospital NHS Trust)

This study will be/has been conducted as part of the portfolio of trials in the registered UKCRC Oxford Clinical Trials Research Unit (OCTRU) at the University of Oxford. It will follow/has followed their Standard Operating Procedures ensuring compliance with the principles of Good Clinical Practice and the Declaration of Helsinki and any applicable regulatory requirements. Special thanks to Nicola Kenealy, Molly Glaze, Gemma Greenall, Rebecca MacLean, Emma Haines, Lucy Eldridge, Juul Achten, Kylea Draper, Elisa Basso, Amrita Athwal and the wider team at the OCTRU and all the physiotherapists, surgeons and research associates at the recruiting centres. Thanks also to Colin Forde for contributing to intervention development.

Contributorship

DJK is the chief investigator and conceived and designed the study, was awarded the funding, and drafted the manuscript. MLC, ET, SH, VSB, SJD, ACR, KW and SEL contributed to study design and provided specific content and edited the manuscript. NP conducted the statistical analysis and SJD oversaw the statistical aspects of the study. VSB provided trial management oversight. All authors have reviewed and approved the manuscript.

Funding statement

This report is independent research supported by the National Institute for Health Research (NIHR Post-Doctoral Fellowship, Dr David Keene, PDF-2016-09-056). The report was supported by the NIHR Biomedical Research Centre, Oxford. Professor Lamb received funding from the NIHR Collaboration for Leadership in Applied Health Research and Care Oxford at Oxford Health NHS Foundation Trust.

For peer review only - http://bmjopen.bmj.com/site/about/guidelines.xhtml

The views expressed in this publication are those of the authors and not necessarily those of the NHS, the National Institute for Health Research, or the Department of Health and Social Care. The study sponsor and funders had no role in study design; writing of the report; and the decision to submit the report for publication.

Competing interests

The institutions of the authors have received research grant funding from NIHR, EU, Royal College of Surgeons England and industry.

Ethics approval

The study was approved by the South Central – Hampshire B Research Ethics Committee (Ref: 18/SC/0281) on 2nd July 2018 and the Health Research Authority and was prospectively registered (ISRCTN16612336).

Data sharing

Data sharing requests can be considered via contact with the corresponding author.

References

1. Court-Brown CM, Caesar B. Epidemiology of adult fractures: a review. *Injury* 2006;37(8):691-97. doi: 10.1016/j.injury.2006.04.130
2. Curtis EM, van der Velde R, Moon RJ, et al. Epidemiology of Fractures in the United Kingdom 1988-2012: Variation with age, sex, geography, ethnicity and socioeconomic status. *Bone* 2016;87:19-26. doi: 10.1016/j.bone.2016.03.006
3. Kannus P, Palvanen M, Niemi S, et al. Stabilizing incidence of low-trauma ankle fractures in elderly people Finnish statistics in 1970-2006 and prediction for the future. *Bone* 2008;43(2):340-2. doi: 10.1016/j.bone.2008.04.015
4. National Institute for Clinical and Care Excellence. Osteoporosis: assessing the risk of fragility fracture Clinical guideline [CG146]2017.
5. Keene DJ, Mistry D, Nam J, et al. The Ankle Injury Management (AIM) Trial: A pragmatic, multi-centre, equivalence randomised controlled trial and economic evaluation comparing close contact casting with open surgical reduction and internal fixation in the treatment of unstable ankle fractures in patients over 60 years. *Health Technol Assess* 2016;20(75):1-158.
6. Willett K, Keene DJ, Mistry D, et al. Close Contact Casting vs Surgery for Initial Treatment of Unstable Ankle Fractures in Older Adults: A Randomized Clinical Trial. *Jama* 2016;316(14):1455-63. doi: 10.1001/jama.2016.14719

For peer review only

For peer review only - http://bmjopen.bmj.com/site/about/guidelines.xhtml

7. Lin CW, Moseley AM, Herbert RD, et al. Pain and dorsiflexion range of motion predict short- and medium-term activity limitation in people receiving physiotherapy intervention after ankle fracture: an observational study. *Aust J Physiother* 2009;55(1):31-7.
8. Psatha M, Wu Z, Gammie FM, et al. A longitudinal MRI study of muscle atrophy during lower leg immobilization following ankle fracture. *J Magn Reson Imaging* 2012;35(3):686-95. doi: 10.1002/jmri.22864
9. Wolf SI, Lessl E, Müller S, et al. Persistent diminished joint function following ankle fracture - a risk factor for posttraumatic osteoarthritis? [abstract]. *Gait Posture* 2013;38 (Suppl 1):S101-02. doi: http://dx.doi.org/10.1016/j.gaitpost.2013.07.207
10. Hancock MJ, Herbert RD, Stewart M. Prediction of outcome after ankle fracture. *J Orthop Sports Phys Ther* 2005;35(12):786-92.
11. Lin CW, Donkers NA, Refshauge KM, et al. Rehabilitation for ankle fractures in adults. *Cochrane Database Syst Rev* 2012;11:Cd005595. doi: 10.1002/14651858.CD005595.pub3
12. Moseley AM, Herbert RD, Nightingale EJ, et al. Passive stretching does not enhance outcomes in patients with plantarflexion contracture after cast immobilization for ankle fracture: a randomized controlled trial. *Arch Phys Med Rehabil* 2005;86(6):1118-26. doi: 10.1016/j.apmr.2004.11.017
13. Lin CW, Moseley AM, Haas M, et al. Manual therapy in addition to physiotherapy does not improve clinical or economic outcomes after ankle fracture. *J Rehabil Med* 2008;40(6):433-9. doi: 10.2340/16501977-0187
14. Nilsson GM, Jonsson K, Ekdahl CS, et al. Effects of a training program after surgically treated ankle fracture: a prospective randomised controlled trial. *BMC Musculoskelet Disord* 2009;10:118. doi: 10.1186/1471-2474-10-118
15. Moseley AM, Beckenkamp PR, Haas M, et al. Rehabilitation After Immobilization for Ankle Fracture: The EXACT Randomized Clinical Trial. *JAMA* 2015;314(13):1376-85. doi: 10.1001/jama.2015.12180
16. Dudek K, Druzbicki M, Przysada G, et al. Assessment of standing balance in patients after ankle fractures. *Acta Bioeng Biomech* 2014;16(4):59-65.
17. Keene DJ, Costa ML, Tutton E, et al. Progressive functional exercise versus best practice advice for adults aged 50 years or over after ankle fracture: protocol for a pilot randomised controlled trial in the UK - the Ankle Fracture Treatment: Enhancing Rehabilitation (AFTER) study. *BMJ Open* 2019;9(11):e030877. doi: 10.1136/bmjopen-2019-030877
18. Kanis JA, Cooper C, Rizzoli R, et al. European guidance for the diagnosis and management of osteoporosis in postmenopausal women. *Osteoporosis international : a journal established as result of cooperation between the European Foundation for Osteoporosis and the National Osteoporosis Foundation of the USA* 2019;30(1):3-44. doi: 10.1007/s00198-018-4704-5
19. Byrne C, Faure C, Keene DJ, et al. Ageing, muscle power, and physical function: A systematic review and implications for pragmatic training interventions. *Sports Med* 2016;In Press
20. National Institute for Clinical and Care Excellence. Falls in older people: Assessing risk and prevention. Manchester: NICE 2013.
21. Kraemer W, Adams K, Cafarelli E, et al. American College of Sports Medicine position stand. Progression models in resistance training for healthy adults. *Med Sci Sports Exerc* 2002;34(2):364-80.
22. Michie S, Rumsey N, Fussell A, et al. Improving health: Changing behaviour.: NHS health trainer handbook, 2008.

For peer review only - http://bmjopen.bmj.com/site/about/guidelines.xhtml

23. Thabane L, Ma J, Chu R, et al. A tutorial on pilot studies: The what, why and how. *BMC Med Res Methodol* 2010;10:1. doi: 10.1186/1471-2288-10-1
24. Olerud C, Molander H. A scoring scale for symptom evaluation after ankle fracture. *Arch Orthop Trauma Surg* 1984;103(3):190-4.
25. Binkley JM, Stratford PW, Lott SA, et al. The Lower Extremity Functional Scale (LEFS): Scale Development, Measurement Properties, and Clinical Application. *Phys Ther* 1999;79(4):371-83. doi: 10.1093/ptj/79.4.371
26. Brooks R. EuroQol: the current state of play. *Health Policy* 1996;37(1):53-72.
27. van Hout B, Janssen MF, Feng YS, et al. Interim scoring for the EQ-5D-5L: mapping the EQ-5D-5L to EQ-5D-3L value sets. *Value Health* 2012;15(5):708-15. doi: 10.1016/j.jval.2012.02.008
28. Kempen GIJM, Yardley L, Van Haastregt JCM, et al. The Short FES-I: a shortened version of the falls efficacy scale-international to assess fear of falling. *Age and Ageing* 2008;37(1):45-50. doi: 10.1093/ageing/afm157
29. Resnick B, Jenkins LS. Testing the Reliability and Validity of the Self-Efficacy for Exercise Scale. *Nurs Res* 2000;49(3):154-59.
30. Bennell KL, Talbot RC, Wajswelner H, et al. Intra-rater and inter-rater reliability of a weight-bearing lunge measure of ankle dorsiflexion. *Aust J Physiother* 1998;44(3):175-80.
31. Wang C-Y, Olson SL, Protas EJ. Test-retest strength reliability: hand-held dynamometry in community-dwelling elderly fallers. *Arch Phys Med Rehabil* 2002;83(6):811-15.
32. Guralnik JM, Simonsick EM, Ferrucci L, et al. A short physical performance battery assessing lower extremity function: Association with self-reported disability and prediction of mortality and nursing home admission. *J Gerontol* 1994;49(2):M85-M94. doi: 10.1093/geronj/49.2.M85
33. Schulz KF, Altman DG, Moher D. CONSORT 2010 statement: updated guidelines for reporting parallel group randomised trials. *BMJ* 2010;340:c332. doi: 10.1136/bmj.c332
34. Eldridge SM, Chan CL, Campbell MJ, et al. CONSORT 2010 statement: extension to randomised pilot and feasibility trials. *BMJ* 2016;355:i5239. doi: 10.1136/bmj.i5239
35. Hoffmann TC, Glasziou PP, Boutron I, et al. Better reporting of interventions: template for intervention description and replication (TIDieR) checklist and guide. *Bmj* 2014;348:g1687. doi: 10.1136/bmj.g1687

For peer review only

For peer review only - http://bmjopen.bmj.com/site/about/guidelines.xhtml

TABLE 1: Intervention descriptions based on TIDieR guidance.

TIDieR Items[35]	Description	
**Brief Name**	AFTER (Ankle Fracture Treatment: Enhancing Rehabilitation)	
**Why**	Physiotherapy-led exercise and advice are commonly used to supervise rehabilitation after ankle fracture, but evidence is lacking in terms of its benefit over advice on self-management.	
	**Progressive exercise**	**Best practice advice**
**What**	Home exercise and advice programme overseen by physiotherapist over≤6 sessions within 16 weeks.	Home exercise and advice programme initiated during a single face-to-face session with a physiotherapist, and then performed unsupervised by participant at home.
**Materials: Participants**	Participant Information Booklet and exercise instruction sheets with photos.<br>Action planner and exercise diary<br>Resistance bands (if applicable)	Participant Information Booklet incorporating exercise instructions with photos.
**Materials: Physiotherapists**	**Therapist training materials:** training session pack detailing study and intervention procedures and a quick reference guide detailing all aspects of the progressive exercise intervention.	**Therapist training materials:** training session pack detailing study and intervention procedures.
**Training**	Up to 4 hours face-to-face training delivered by AFTER trial research physiotherapist.<br>Training pack detailing all aspects of the trial and both interventions.	
**Procedures**	**Initial appointment**<br>Assess participant as per normal physiotherapy practice.<br>Issue folder containing the Progressive Exercise Participant Information Booklet.<br>Agree level of exercise that is most appropriate for the participant initially.<br>Advice should address barriers to exercise identified during assessment.<br>Provide education regarding pain during and after exercise.<br>Help participant to complete exercise documentation (exercise diary and action planner).<br>Make follow-up appointment(s).<br>Complete Treatment Log.<br><br>**Appointments 2-6**<br>Reassess as per normal physiotherapy practice.<br>Assist participant to progress/regress exercises.<br>Reassure the participant, reinforce key messages from the advice and education.<br>Review home exercise programme using the exercise diary.<br>Discuss return to functional activities.<br>Review action planner.<br>Complete Treatment Log (after every session).	**Single face-to-face appointment**<br>Assess participant as per normal physiotherapy practice<br>Issue Best Practice Advice Participant Information Booklet.<br>Explain exercise difficulty level to start on based on assessment.<br>Educate participants how to progress and regress their exercises.<br>Advice should address barriers to exercise identified during assessment.<br>Provide education regarding pain during and after exercise.<br>Discharge participant with advice/encouragement to continue with the self-management exercise programme for at least 16 weeks.<br>Complete Treatment Log.<br><br>**Up to two further advice sessions (optional for participants having difficulties with self-management or the exercises)**<br><br>Physiotherapist's role was to re-assess and re-enforce self-management advice.

For peer review only - http://bmjopen.bmj.com/site/about/guidelines.xhtml

**Who provides**	Physiotherapists already working in NHS musculoskeletal services who have attended the progressive exercise intervention training.<br>AFTER did not exclude physiotherapists based on number of years qualified or experience.	Physiotherapists already working in NHS musculoskeletal services who have attended the best practice advice intervention training.<br>AFTER did not exclude physiotherapists based on number of years qualified or experience.
**How**	Participants receive up to 6 sessions with a physiotherapist over 16 weeks.<br>The initial session 20 to 60 minutes for assessment. It is then followed by up to five sessions of up to 30 minutes each.	Participants receive a single face-to-face session with a physiotherapist lasting 20 to 60 minutes.<br>Up to two further advice sessions were optional.
**Where**	Physiotherapy sessions were in outpatient clinics based in the UK NHS.<br>Exercise programme is performed by the participant at home.	Same as progressive exercise intervention.
**When and how much**	Initial appointment was as soon as possible after splint/cast removal, as per local appointment availability.<br>Up to 5 follow-up sessions arranged within 16 weeks.<br>Could be less than 6 sessions if participant has met rehabilitation goals and was self-managing condition.	The initial appointment was the same as progressive exercise in terms of timing, but additional one or two sessions were optional for participants that were having difficulties with self-management or the exercises.
**Tailoring**	**Education and advice**<br>Focus of education and advice were individualised based on assessment.<br>**Exercises**<br>Selection, manipulation of sets, repetitions and/or load is a joint decision-making process.<br>Range of motion and position could be modified to accommodate the patient's comfort and preferences.	**Education and advice**<br>Focus of education and advice are individualised based on assessment.<br>**Exercises**<br>The range of motion through which an exercise is performed, and the load and volume, could be increased or decreased.
**Modifications**	The optional follow-up sessions for best practice advice were initially set as face to face or telephone. Later in recruitment this was altered to telephone only.	
**Intervention Fidelity**		
**How well: <u>Training</u>**	All aspects of training delivery, content, structure, duration and therapists' confidence to implement the intervention were evaluated using post training feedback forms completed anonymously.	
**How well: <u>Physiotherapists</u>**	Intervention fidelity was monitored centrally via treatment logs, and during site visits.	
**How well: <u>Participants</u>**	**Exercise adherence**<br>Physiotherapists review the exercise diary at each subsequent session.<br>Participants asked to report exercise frequency in postal follow-up questionnaires at three and six months.	**Exercise adherence**<br>Participants asked to report exercise frequency in postal follow-up questionnaires at three and six months.
**How well: <u>Reporting</u>**	Intervention delivery data captured on treatment logs was entered onto the trial database and the findings are reported in the results section of this manuscript.	

For peer review only - http://bmjopen.bmj.com/site/about/guidelines.xhtml

TABLE 2: Baseline characteristics

	Best Practice Advice	Progressive Exercise	Total
Age (mean(SD))	63.3 (7.2)	63.7 (10.4)	63.5 (8.9)
Sex (n (%))			
Female	22 (73.3%)	20 (64.5%)	42 (68.9%)
Male	8 (26.7%)	11 (35.5%)	19 (31.1%)
BMI [1]	27.9 (4.8)	29.0 (6.8)	28.5 (5.9)
Ethnicity (n (%))			
Black or black British	1 (3.3%)	2 (6.5%)	3 (4.9%)
Mixed	1 (3.3%)	1 (3.2%)	2 (3.3%)
Other	1 (3.3%)	0 (0%)	1 (1.6%)
White British	27 (90%)	26 (83.9%)	53 (86.9%)
White other	0 (0%)	2 (6.5%)	2 (3.3%)
Education (n (%))			
Higher professional or university education	17 (56.7%)	14 (45.2%)	31 (50.8%)
None or Primary education	3 (10%)	0 (0%)	3 (4.9%)
Secondary education	10 (33.3%)	17 (54.8%)	27 (44.3%)
Smoking status (n (%))			
Current smoker	3 (10%)	4 (12.9%)	7 (11.5%)
Former smoker	10 (33.3%)	15 (48.4%)	25 (41%)
Never smoked	17 (56.7%)	12 (38.7%)	29 (47.5%)
If current smoker, number of cigarettes per day (mean (SD))	9.3 (6.9)	17.5 (17.9)	14.2 (14.9)
Alcohol units per week (mean (SD))	11.1 (13.4)	5.5 (9.1)	8.2 (11.6)
Employment status (n (%))			
Employed	11 (36.7%)	14 (45.2%)	25 (41%)
Other	1 (3.3%)	0 (0%)	1 (1.6%)
Permanently sick or disabled	0 (0%)	1 (3.2%)	1 (1.6%)
Retired	14 (46.7%)	14 (45.2%)	28 (45.9%)
Semi-retired	4 (13.3%)	1 (3.2%)	5 (8.2%)
Unemployed	0 (0%)	1 (3.2%)	1 (1.6%)
Time off work (due to injury) (n (%))			
Not in paid work	13 (43.3%)	15 (48.4%)	28 (45.9%)
No	4 (13.3%)	3 (9.7%)	7 (11.5%)
Yes	12 (40%)	12 (38.7%)	24 (39.3%)
Work days missed (mean (SD))	16.1 (28.4)	19.3 (19.1)	17.9 (23.2)
Accommodation type (n (%))			
House/flat rented from housing association/local authority	3 (10%)	3 (9.7%)	6 (9.8%)
Other	2 (6.7%)	0 (0%)	2 (3.3%)
Owner occupied house/flat	21 (70%)	21 (67.7%)	42 (68.9%)
Privately rented house/flat	3 (10%)	7 (22.6%)	10 (16.4%)
Sheltered housing/warden control	1 (3.3%)	0 (0%)	1 (1.6%)
Unpaid carer (n (%))			
Yes	5 (16.7%)	9 (29.0%)	14 (23.0%)
No	25 (83.3%)	22 (71.0%)	47 (77.0%)
Resident unpaid carer (n (%))			
Yes	4 (13.3%)	5 (16.1%)	9 (14.8%)
No	1 (3.3%)	4 (12.9%)	5 (8.2%)
No unpaid carer	25 (83.3%)	22 (71.0%)	47 (77.0%)
Paid carer (n (%))			
Yes	2 (6.7%)	2 (6.5%)	4 (6.6%)
No	28 (93.3%)	28 (90.3%)	56 (91.8%)
Unknown/Missing	0 (0%)	1 (3.2%)	1 (1.6%)
Resident paid carer (n (%))			
Yes	1 (3.3%)	2(6.5%)	3 (4.9%)
No	1 (3.3%)	0 (0%)	1 (1.6%)
No paid carer	28 (93.3%)	28 (90.3%)	56 (91.8%)
Unknown/Missing	0 (0%)	1 (3.2%)	1 (1.6%)
Treatment type (n (%))			
Conservative (non-surgical) management	15 (50.0%)	13 (41.9%)	28 (45.9%)

For peer review only - http://bmjopen.bmj.com/site/about/guidelines.xhtml

	Best Practice Advice	Progressive Exercise	Total
Trad. Cast non-removable	3 (10.0%)	0 (0%)	3 (4.9%)
Trad. Cast removable	1 (3.3%)	3 (9.7%)	4 (6.6%)
Close contact casting	5 (16.7%)	4 (12.9%)	9 (14.8%)
Walking boot	6 (20.0%)	6 (19.4%)	12 (19.7%)
Unknown/Missing	3 (10.0%)	3 (9.7%)	6 (9.8%)
Surgical management	12 (40.0%)	15 (48.4%)	27 (44.3%)
Clinical Outcomes (mean (SD))			
OMAS[2]	31.38 (17.97)	21.50 (15.71)	26.36 (17.44)
LEFS[3]	25.07 (13.39)	17.52 (11.68)	21.10 (12.98)
FES-I[4]	14.75 (5.18)	17.00 (6.16)	15.85 (5.74)
EQ-5D Utility[5]	0.48 (0.29)	0.25 (0.30)	0.36 (0.31)
EQ-5D VAS[6]	69.52 (19.11)	68.63 (20.04)	69.07 (19.42)
Pain at rest VAS[7]	26.6 (28.2)	32.1 (27.5)	29.4 (27.7)
Pain walking VAS[7]	37.8 (30.0)	55.2 (27.0)	46.2 (29.6)
Falls (last 3 months)	14.8 (5.2)	17.0 (6.2)	15.9 (5.7)
Falls that resulted in a broken bone (last 3 months) (n (%))			
No	12 (40%)	10 (32.3%)	22 (36.1%)
Yes[8]	2 (6.7%)	3 (9.7%)	5[1] (8.2%)
Unknown/Missing	16 (53.3%)	18 (58.1%)	34 (55.7%)
Aids pre-injury (n (%))			
Frame/rollator	8 (26.7%)	1 (3.2%)	2 (3.3%)
None	5 (16.7%)	27 (87.1%)	51 (83.6%)
One crutch	3 (10%)	1 (3.2%)	1 (1.6%)
One stick	3 (10%)	0 (0%)	3 (4.9%)
Two crutches	8 (26.7%)	1 (3.2%)	1 (1.6%)
Two sticks	1 (3.3%)	1 (3.2%)	1 (1.6%)
Unknown/Missing	2 (6.7%)	0 (0%)	2 (3.3%)
Aids since injury (n (%))			
Frame/rollator	8 (26.7%)	13 (41.9%)	21 (34.4%)
None	5 (16.7%)	1 (3.2%)	6 (9.8%)
One crutch	3 (10%)	1 (3.2%)	4 (6.6%)
One stick	3 (10%)	0 (0%)	3 (4.9%)
Two crutches	8 (26.7%)	11 (35.5%)	19 (31.1%)
Two sticks	1 (3.3%)	2 (6.5%)	3 (4.9%)
Unknown/Missing	2 (6.7%)	3 (9.7%)	5 (8.2%)
Walking distance pre-injury (n (%))			
About the house	1 (3.3%)	2 (6.5%)	3 (4.9%)
Less than half a mile	3 (10%)	4 (12.9%)	7 (11.5%)
More than half a mile	24 (80%)	25 (80.6%)	49 (80.3%)
Bedbound	0 (0%)	0 (0%)	0 (0%)
Unknown/Missing	2 (6.7%)	0 (0%)	2 (3.3%)
Medication Use (n (%))			
Yes	21 (70%)	27 (87.1%)	48 (78.7%)
No	9 (30%)	4 (12.9%)	13 (21.3%)

1 BMI calculated as height divided by weight squared (m/kg^2)
2 OMAS: Olerud and Molander Ankle Score (0-100, higher scores better)
3 LEFS: Lower Extremity Functional Scale (0-100, higher scores better)
4 FES-I: Falls Efficacy Scale-International (short version): scores range from 7 (no concern about falling) to a maximum of 28 (severe concern about falling)
5 EQ-5D-5L utility, -0.594, indicating the worst possible health state, to 1.0 and is anchored at 0 [death] and 1.0 full health]
6 EQ-VAS gives self-rated health on a scale where the endpoints are labelled from 'worst imaginable health state' (0) to 'best imaginable health state' (100)
7 VAS: visual analogue scale, 0-100 scale, higher scores worse
8 Excluding index ankle fracture

For peer review only - http://bmjopen.bmj.com/site/about/guidelines.xhtml

TABLE 3: Compliance with the allocated treatment by trial arm

	Best Practice Advice	Progressive Exercise
**Totals**	**N = 30**	**N = 31**
Completed exercise treatment[1], n (%)	28 (93.3%)	18 (58.1%)
Partial exercise completion[2], n (%)	28 (93.3%)	28 (90.3%)
Received no treatment, n (%)	2 (6.7%)	3 (7.1%)
DNA/unable to contact	0 (0%)	1 (3.2%)
Withdrawal/declined	0 (0%)	0 (0%)
Too difficult to attend appointments	0 (0%)	1 (3.2%)
Unknown	2 (6.7%)	1 (3.2%)
**Median number of sessions (IQR)**	**1 (1, 2)**	**5 (3, 6)**
1 Sessions, n	20	3
2 Sessions, n	5	2
3 Sessions, n	-	3
4 Sessions, n	2	5
5 Sessions, n	1	7
6 Sessions, n	-	7
7 Sessions, n	-	1
Participants receiving additional sessions [3], n (%)	8 (26.7%)	1 (3.2%)
Before protocol change [4]	3	1
After protocol change [4]	5	0
Total number of additional contact sessions, n	13	1
Telephone	2	0
Face-to-face	11	1

[1] Best practice advice: attendance at ≥1 session; Progressive exercise: 6 sessions attended, or discharged by clinician as treatment completed (as marked on treatment log), or discharged by clinician following patient-initiated follow-up period with no further contact, or referred on for further investigation/treatment

[2] Defined as attendance at ≥1 session

[3] Additional sessions defined as >1 for Best Practice Advice, and >6 for progressive exercise

[4] All the extra sessions for the Best Practice Advice arm before the protocol change were face-to-face, of the five participants who got additional sessions after the protocol change, 2 of them were by telephone, and 3 were face-to-face

For peer review only

TABLE 4: Intervention delivery

Treatment components, N (%[1])	Best Practice Advice only<br>N (received BPA) = 28	Progressive exercise<br>N (received at least one session) = 28
Exercises prescribed	27 (96.4%)	23 (82.1%)
Information booklet provided	28 (100%)	24 (85.7%)
Session time in minutes (median, IQR)	45 (40, 60)	First session: 45 (45, 40)<br>Follow-up sessions: 30 (30, 45)

For peer review only

TABLE 5: Summary statistics for each clinical outcome score

	Best Practice Advice			Progressive Exercise			Mean difference (95% CIs)	
	n	mean	SD	n	mean	SD	Mean difference	95% CIs
**OMAS[1]**								
Baseline	29	31.38	17.97	30	21.50	15.71		
Month 3	28	65.54	20.29	26	62.50	23.59		
Month 6	28	73.39	22.07	26	77.12	21.73	1.49	(-6.87, 9.86)
Change from baseline	27	43.70	14.58	25	55.00	24.87	-11.30	(-22.55, -0.04)
**LEFS[2]**								
Baseline	28	25.07	13.39	31	17.52	11.68		
Month 3	26	54.77	12.85	26	57.38	17.41		
Month 6	25	65.36	11.45	27	61.52	20.05	1.02	(-6.28, 8.32)
Change from baseline	23	40.57	12.78	27	44.59	20.17	-4.03	(-13.83, 5.77)
**FES-I[3]**								
Baseline	28	14.75	5.18	27	17.00	6.16		
Month 3	25	10.04	3.22	26	9.65	3.72		
Month 6	26	9.35	3.02	26	9.81	5.02	-0.62	(-2.08, 0.83)
Change from baseline	26	-5.31	5.12	23	-8.30	5.34	3.00	(-0.011, 6.00)
**EQ-5D Utility[4]**								
Baseline	29	0.48	0.29	30	0.25	0.30		
Month 3	27	0.72	0.20	25	0.74	0.14		
Month 6	28	0.77	0.15	28	0.81	0.27	-0.014	(-0.108, 0.080)
Change from baseline	27	0.30	0.31	27	0.57	0.35	-0.27	(-0.45, -0.09)
**EQ-5D VAS[5]**								
Baseline	29	69.52	19.11	30	68.63	20.04		
Month 3	27	74.37	22.93	26	83.27	15.53		
Month 6	28	82.07	17.85	28	81.36	18.21	2.03	(-3.53, 7.58)
Change from baseline	28	11.56	15.01	28	14.04	21.71	1.47	(-4.26, 7.20)
**SPPB[6]**								
Month 6	22	10.27	1.91	25	10.24	1.59	0.12	(-0.82, 1.05)
Month 6[7]	27	8.48	4.41	28	9.36	3.60	0.98	(-1.24, 3.20)
**Range of Motion (RoM) at 6 months (°)**								
dorsiflexion (uninjured)	24	10.8	5.4	26	12.5	8.7		
dorsiflexion (injured)	24	9.2	5.1	26	7.6	9.0		
dorsiflexion (difference)	24	1.7	3.3	26	4.9	8.1		
**% RoM dorsal flexion[8]**		**85.2%**			**60.8%**		3.55	(-1.03, 7.20)
plantarflexion (uninjured)	24	47.8	17.0	26	43.5	14.7		
plantarflexion (injured)	24	43.0	16.2	26	38.0	12.2		
plantarflexion (difference)	24	4.8	10.1	26	5.5	7.0		

For peer review only - http://bmjopen.bmj.com/site/about/guidelines.xhtml

	Best Practice Advice			Progressive Exercise			Mean difference (95% CIs)	
	n	mean	SD	n	mean	SD	Mean difference	95% CIs
**% RoM plantar flexion[8]**		**90.0%**			**87.4%**		**0.22**	**(-4.71, 5.14)**
inversion (uninjured)	24	33.5	10.1	26	31.5	10.8		
inversion (injured)	24	26.0	10.7	26	26.3	11.7		
inversion (difference)	24	7.5	8.0	26	5.2	8.7		
**% RoM inversion[8]**		**77.6%**			**83.5%**		**-3.19**	**(-7.49, 1.10)**
eversion (uninjured)	23	18.5	6.6	26	17.1	6.3		
eversion (injured)	23	15.0	5.6	26	15.6	7.5		
eversion (difference)	23	3.5	5.5	26	1.5	6.3		
**% RoM eversion[8]**		**81.2%**			**91.2%**		**-1.70**	**(-5.24, 1.84)**
**Strength[9] at 6 months (Newtons)**								
Dorsiflexion strength	24	151.3	54.4	26	174.2	99.9	32.24	(2.85, 61.64)
Plantarflexion strength	24	221.1	85.2	26	212.4	107.8	6.34	(-28.83, 41.50)
**Pain at rest VAS[10]**								
Baseline	29	26.6	28.2	31	32.1	27.5		
Month 3	26	11.6	18.1	26	16.8	24.0		
Month 6	25	6.5	13.6	28	8.2	22.7	-0.82	(-6.64, 5.01)
**Pain walking VAS[10]**								
Baseline	28	37.8	30.0	26	55.2	27.0		
Month 3	27	19.1	23.5	25	19.6	24.1		
Month 6	26	12.2	19.6	27	10.7	22.8	-1.11	(-9.61, 7.40)
**Self-Efficacy for Exercise Scale[11]**								
Month 3	18	61.7	27.2	20	71.0	22.0		
Month 6	23	69.6	21.2	25	67.8	21.5	2.24	(-12.11, 16.60)

1 OMAS: Olerud and Molander Ankle Score (0-100, higher scores better)
2 LEFS: Lower Extremity Functional Scale (0-100, higher scores better)
3 FES-I: Falls Efficacy Scale-International (short version), scores range from 7 (no concern about falling) to a maximum of 28 (severe concern about falling).
4 EQ-5D-5L utility, -0.594, indicating the worst possible health state, to 1.0 and is anchored at 0 [death] and 1.0 full health].
5 EQ-VAS gives self-rated health on a scale where the endpoints are labelled from 'worst imaginable health state' (0) to 'best imaginable health state' (100).
6 SPPB: Short Physical Performance Battery (scores range from 0 to 12, higher scores better).
7 Imputes 0 for those participants who completed form, but left questions blank – they were assumed to have not attempted
8 Calculated using the injured divided by the uninjured scores, multiplied by 100 to output as a percentage
9 The highest strength score (measured in Newtons N) out of 3 was used for each patient
10 VAS: visual analogue scale (0-100 scale, higher scores worse)
11 Self-efficacy exercise score. Overall scores range 0 to 90, with higher scores indicating greater confidence to exercise.

For peer review only - http://bmjopen.bmj.com/site/about/guidelines.xhtml

TABLE 6: Feasibility assessment

	Target	Outcome
Participation rate	A study participation rate of at least 25% of those eligible, to indicate acceptability and generalisability.	Sixty-one of 112 (54%) eligible participants participated in the trial, exceeding the feasibility criteria for participation of 25%.
Recruitment	At least 48 eligible participants across at least three sites agreed to participate over a maximum of 18 months.	61 participants were recruited in 11 months at 5 centres. There were more sites than originally planned so to interpret this feasibility criteria the recruitment rate per centre month was more appropriate. The recruitment feasibility criteria were based on 1.5 participants per site per month. 1.4 participants per centre month was achieved during the pilot trial.
Completion of study intervention sessions	At least 85% of participants completed study intervention sessions.	At least one intervention was provided for 28/30 (93%) best practice advice and 28/31 (90%) progressive exercise participants. 18/31 (58%) participants met the criteria for fully completing the progressive exercise intervention.
Follow-up rate	At least 80% of participants attended study follow-up at 6 months.	Defined as the number who provided OMAS scores at 6 months, was 28/30 (93%) for the best practice advice group and 26/31 (84%) in the progressive exercise group, an overall follow-up rate of 89%.

For peer review only

Figure legends:

Figure 1: Flow of participants through the AFTER pilot trial

[1] One site has missing screening data for some of the randomised participants, so those who were randomised were added to the totals screened
[2] Major ankle trauma defined as: Open fracture wounds, external fixation, or substantial skin loss or grafts, that would limit ankle or lower leg exercise
[3] Compliance is defined as attending at least one session
*[4] Loss to follow-up is defined as **no response** at the 6 month follow-up*
[5] OMAS scores at 6 months

For peer review only

For peer review only - http://bmjopen.bmj.com/site/about/guidelines.xhtml

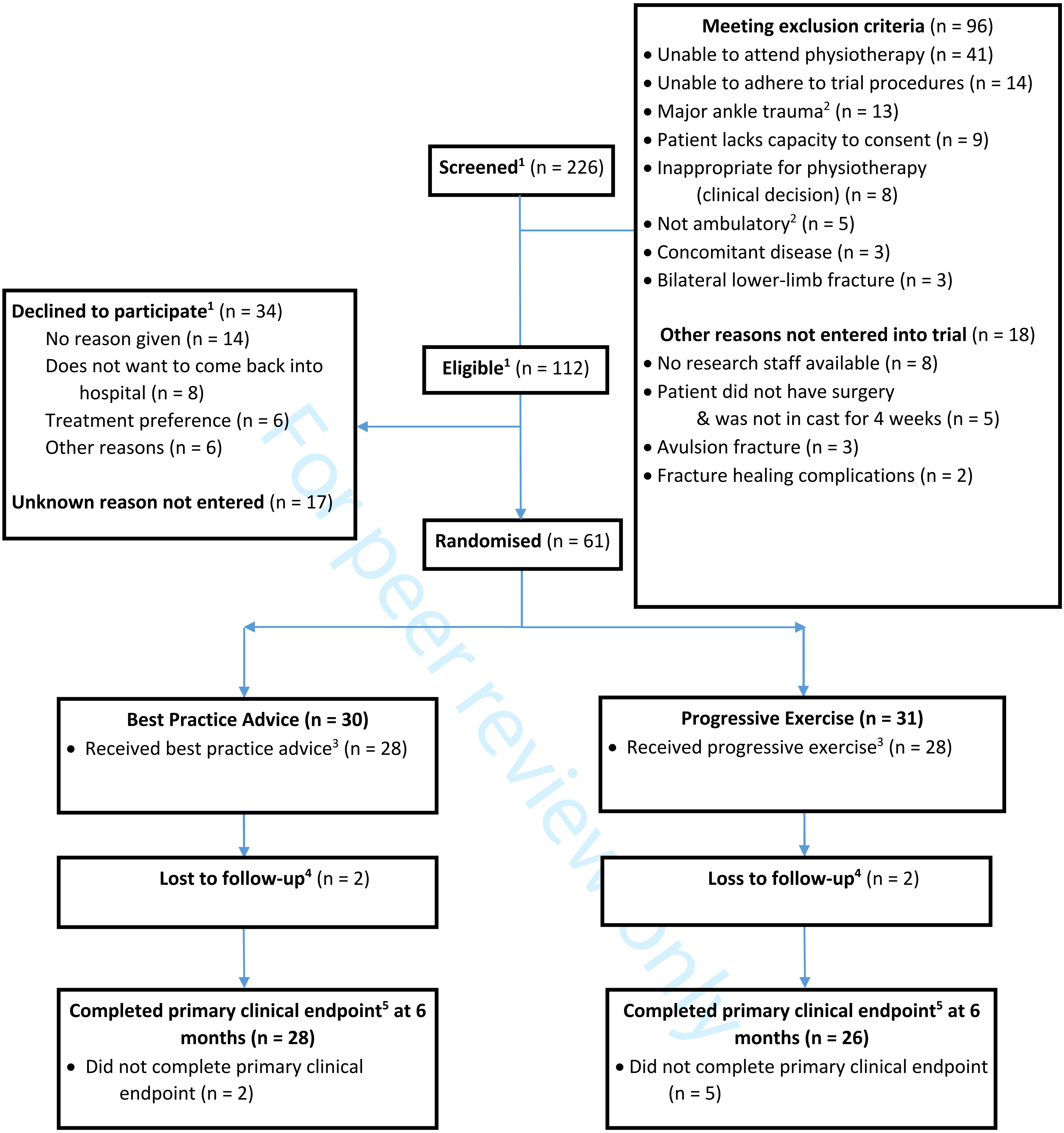

For peer review only

Supplementary appendix

Table S1: *Health resource use at 3 months*

	Best practice advice		Progressive exercise		Total	
	n	%	n	%	n	%
**Pain increase**						
Doctor seen	0	0%	1	3.2%	1	1.6%
Painkillers	3	10%	2	6.5%	5	8.2%
Further pain	1	3.3%	0	0%	1	1.6%
**Condition worsened**						
Doctor seen	2	6.7%	1	3.2%	3	4.9%
Further treatment	1	3.3%	1	3.2%	2	3.3%
Further worsening	1	3.3%	0	0%	1	1.6%
**Prescription medications**						
Yes	7	23.3%	13	41.9%	20	32.8%
No	23	76.7%	18	58.1%	41	67.2%
**Over the counter medications**						
Yes	10	33.3%	7	22.6%	17	27.9%
No	20	66.7%	24	77.4%	44	72.1%
**NHS community services**						
GP visit	3	10%	2	6.5%	5	8.2%
GP telephone	0	0%	1	3.2%	1	1.6%
Practice nurse	0	0%	2	6.5%	2	3.3%
Physio at hospital	3	10%	1	3.2%	4	6.6%
Community health team	1	3.3%	0	0%	1	1.6%
Consultant for extra scan	1	3.3%	0	0%	1	1.6%
Other	1	3.3%	0	0%	1	1.6%
**NHS outpatient**						
Orthopaedic clinic	9	30%	6	19.4%	15	24.6%
Physiotherapy department	3	10%	0	0%	3	4.9%
X-ray	4	13.3%	2	6.5%	6	9.8%
Ultrasound	1	3.3%	0	0%	1	1.6%
A & E	2	6.7%	2	6.5%	4	6.6%
Other outpatient	4	13.3%	3	9.7%	7	11.5%
**NHS inpatient**						
Broken ankle	0	0%	1	3.2%	1	1.6%
**Private outpatient**						
Orthopaedic clinic	1	3.3%	1	3.2%	2	3.3%
Physiotherapy department	2	6.7%	1	3.2%	3	4.9%
Other outpatient	2	6.7%	0	0%	2	3.3%
**Expenses occurred**						
Yes	7	23.3%	19	61.3%	26	42.6%
No	15	50%	7	22.6%	22	36.1%
Unknown	8	26.7%	5	16.1%	13	21.3%
**Expenses breakdown**						
Travel and taxi fares	7	23.3%	6	19.4%	13	21.3%

For peer review only

	Best practice advice		Progressive exercise		Total	
	n	%	n	%	n	%
Parking	2	6.7%	2	6.5%	2	3.3%
Care costs	2	6.7%	2	6.5%	2	3.3%
Other	6	20%	4	12.9%	10	16.4%

Table S2: *Health resource use at 6 months*

	Best practice advice		Progressive exercise		Total	
	n	%	n	%	n	%
**Condition worsened**						
Doctor seen	1	3.3%	0	0%	1	1.6%
Further treatment	1	3.3%	0	0%	1	1.6%
**Prescription medications**						
Yes	4	13.3%	9	29.0%	13	21.3%
No	26	86.7%	22	71.0%	48	78.7%
**Over the counter medications**						
Yes	5	16.7%	2	6.5%	7	11.5%
No	25	83.3%	29	93.5%	54	88.5%
**NHS community services**						
GP visit	2	6.7%	3	9.7%	5	8.2%
Physio at home	0	0%	1	3.2%	1	1.6%
Physio at home	0	0%	1	3.2%	1	1.6%
**NHS outpatient**						
Orthopaedic clinic	0	0%	1	3.2%	1	1.6%
Physiotherapy department	1	3.3%	0	0%	1	1.6%
A & E	1	3.3%	0	0%	1	1.6%
**NHS inpatient**						
Unknown reason	0	0%	1	3.2%	1	1.6%
**Private outpatient**						
Physiotherapy department	2	6.7%	1	3.2%	3	4.9%
Other outpatient	2	6.7%	1	3.2%	3	4.9%
**Expenses occurred**						
Yes	2	6.7%	3	9.7%	5	8.2%
No	23	76.7%	23	74.2%	26	42.6%
Unknown	5	16.7%	5	16.1%	10	16.4%
**Expenses breakdown**						
Travel and taxi fares	0	0%	3	9.7%	3	4.9%
Parking	2	6.7%	0	0%	2	3.3%
Other	1	3.3%	4	12.9%	5	8.2%

For peer review only

Table S3: return to desired activities, including work, social life, and sport activities, walking aid use and distances at three months

	Best practice advice		Progressive exercise		Total	
	n	%	n	%	n	%
**Walking aids**						
Frame/rollator	1	3.3%	1	3.2%	2	3.3%
None	20	66.7%	20	64.5%	40	65.6%
One crutch	2	6.7%	1	3.2%	3	4.9%
One stick	3	10%	2	6.5%	5	8.2%
Two crutches	1	3.3%	1	3.2%	2	3.3%
Two sticks	0	0%	1	3.2%	1	1.6%
Missing	3	10%	5	16.1%	8	13.1%
**Walking distance possible**						
About the house	0	0%	2	6.5%	2	3.3%
Less than 100 metres	2	6.7%	2	6.5%	4	6.6%
Less than half a mile	4	13.3%	4	12.9%	8	13.1%
More than half a mile	21	70%	18	58.1%	39	63.9%
Missing	3	10%	5	16.1%	8	13.1%
**Recreational activities**						
Missing	5	16.7%	5	16.1%	10	16.4%
No difficulty	4	13.3%	8	25.8%	12	19.7%
Mild difficulty	11	36.7%	9	29%	20	32.8%
Moderate difficulty	4	13.3%	6	19.4%	10	16.4%
Severe difficulty	5	16.7%	0	0%	5	8.2%
Unable	1	3.3%	3	9.7%	4	6.6%
**Family/friend interference**						
Missing	4	13.3%	5	16.1%	9	14.8%
Not at all	12	40%	14	45.2%	26	42.6%
Slightly	8	26.7%	7	22.6%	15	24.6%
Moderately	2	6.7%	3	9.7%	5	8.2%
Quite a bit	3	10%	1	3.2%	4	6.6%
Extremely	1	3.3%	1	3.2%	2	3.3%
**Daily activities**						
Missing	4	13.3%	5	16.1%	9	14.8%
Not at all limited	11	36.7%	15	48.4%	26	42.6%
Slightly limited	7	23.3%	5	16.1%	12	19.7%
Moderately limited	7	23.3%	5	16.1%	12	19.7%
Very limited	1	3.3%	0	0%	1	1.6%
Extremely limited	0	0%	1	3.2%	1	1.6%
**Improvement[1]**						
-1	1	3.3%	1	3.2%	2	3.3%
-2	1	3.3%	0	0%	1	1.6%
-3	0	0%	1	3.2%	1	1.6%
0	0	0%	4	12.9%	4	6.6%
1	0	0%	2	6.5%	2	3.3%
2	1	3.3%	3	9.7%	4	6.6%
3	12	40%	6	19.4%	18	29.5%
4	6	20%	6	19.4%	12	19.7%
5	2	6.7%	3	9.7%	5	8.2%

For peer review only - http://bmjopen.bmj.com/site/about/guidelines.xhtml

	Best practice advice		Progressive exercise		Total	
	n	%	n	%	n	%
Missing	7	23.3%	5	16.1%	12	19.7%
**Doing Exercises**						
Missing	7	23.3%	6	19.4%	13	21.3%
No	3	10%	4	12.9%	7	11.5%
Yes	20	66.7%	21	67.7%	41	67.2%
**Exercise frequency (days per week)**						
Missing	10	33.3%	10	32.3%	20	32.8%
2	1	3.3%	0	0%	1	1.6%
3	3	10%	1	3.2%	4	6.6%
5	5	16.7%	5	16.1%	10	16.4%
6	2	6.7%	1	3.2%	3	4.9%
7	9	30%	14	45.2%	23	37.7%
**Employment**						
Missing	6	20%	5	16.1%	11	18%
No	15	50%	14	45.2%	29	47.5%
Yes	9	30%	12	38.7%	21	34.4%
**Time-off due to ankle injury**						
Missing	21	70%	20	64.5%	41	67.2%
No	4	13.3%	6	19.4%	10	16.4%
Yes	5	16.7%	5	16.1%	10	16.4%

[1] *Scale ranges from -5 denoting 'very much worse' to 5 denoting 'completely recovered', and with 0 denoting 'unchanged'*

Table S4: return to desired activities, including work, social life, and sport activities, walking aid use and distances at six months

	Best practice advice		Progressive exercise		Total	
	n	%	n	%	n	%
**Walking aids**						
Frame/rollator	1	3.3%	1	3.2%	2	3.3%
None	22	73.3%	24	77.4%	46	75.4%
One stick	4	13.3%	2	6.5%	6	9.8%
Two crutches	0	0%	1	3.2%	1	1.6%
Missing	3	10%	3	9.7%	6	9.8%
**Walking distance possible**						
Bedbound	0	0%	1	3.2%	1	1.6%
About the house	0	0%	1	3.2%	1	1.6%
Less than 100 metres	3	10%	1	3.2%	4	6.6%
Less than half a mile	2	6.7%	2	6.5%	4	6.6%
More than half a mile	22	73.3%	23	74.2%	45	73.8%
Missing	3	10%	3	9.7%	6	9.8%
**Recreational activities**						
Missing	3	10%	4	12.9%	7	11.5%

	Best practice advice		Progressive exercise		Total	
	n	%	n	%	n	%
No difficulty	13	43.3%	18	58.1%	31	50.8%
Mild difficulty	9	30%	6	19.4%	15	24.6%
Moderate difficulty	2	6.7%	2	6.5%	4	6.6%
Severe difficulty	2	6.7%	0	0%	2	3.3%
Unable	1	3.3%	1	3.2%	2	3.3%
**Family/friend interference**						
Missing	3	10%	4	12.9%	7	11.5%
Not at all	19	63.3%	21	67.7%	40	65.6%
Slightly	6	20%	2	6.5%	8	13.1%
Moderately	1	3.3%	1	3.2%	2	3.3%
Quite a bit	0	0%	2	6.5%	2	3.3%
Extremely	1	3.3%	1	3.2%	2	3.3%
**Daily activities**						
Missing	3	10%	4	12.9%	7	11.5%
Not at all limited	17	56.7%	20	64.5%	37	60.7%
Slightly limited	8	26.7%	5	16.1%	13	21.3%
Moderately limited	2	6.7%	0	0%	2	3.3%
Very limited	0	0%	1	3.2%	1	1.6%
Extremely limited	0	0%	1	3.2%	1	1.6%
**Improvement[1]**						
-1	0	0%	3	9.7%	3	4.9%
-3	2	6.7%	0	0%	2	3.3%
-4	0	0%	1	3.2%	1	1.6%
Missing	3	10%	4	12.9%	7	11.5%
0	1	3.3%	1	3.2%	2	3.3%
2	1	3.3%	3	9.7%	4	6.6%
3	4	13.3%	2	6.5%	6	9.8%
4	13	43.3%	9	29%	22	36.1%
5	6	20%	8	25.8%	14	23%
**Doping Exercises**						
Missing	4	13.3%	5	16.1%	9	14.8%
No	12	40%	7	22.6%	19	31.1%
Yes	14	46.7%	19	61.3%	33	54.1%
**Exercise frequency (days per week)**						
Missing	17	56.7%	12	38.7%	29	47.5%
1	0	0%	1	3.2%	1	1.6%
2	1	3.3%	1	3.2%	2	3.3%
3	2	6.7%	6	19.4%	8	13.1%
4	2	6.7%	3	9.7%	5	8.2%
5	2	6.7%	3	9.7%	5	8.2%
6	0	0%	1	3.2%	1	1.6%
7	6	20%	4	12.9%	10	16.4%
**Employment**						
Missing	2	6.7%	4	12.9%	6	9.8%
No	18	60%	14	45.2%	32	52.5%
Yes	10	33.3%	13	41.9%	23	37.7%
**Time-off due to ankle injury**						
Missing	20	66.7%	18	58.1%	38	62.3%

For peer review only

	Best practice advice		Progressive exercise		Total	
	n	%	n	%	n	%
No	9	30%	11	35.5%	20	32.8%
Yes	1	3.3%	2	6.5%	3	4.9%

[1] Scale ranges from -5 denoting 'very much worse' to 5 denoting 'completely recovered', and with 0 denoting 'unchanged'

For peer review only

Table S5: Adverse events summarised at 3 and 6 months

	Best practice advice		Progressive exercise		Total	
	n	%	n	%	n	%
**3 Months**						
**Recent falls (last 3 months)**						
No	22	73.3%	22	71%	44	72.1%
Yes	4	13.3%	4	12.9%	8	13.1%
Missing	4	13.3%	5	16.1%	9	14.8%
**Number of recent falls**						
0	26	86.7%	27	87.1%	53	86.9%
1	2	6.7%	3	9.7%	5	8.2%
2	1	3.3%	0	0%	1	1.6%
3	1	3.3%	0	0%	1	1.6%
6	0	0%	1	3.2%	1	1.6%
**Broken bones (from recent fall, if applicable)**						
Yes	0	0%	0	0%	0	0%
No	4	13.3%	4	12.9%	8	13.1%
**Pain increase**						
Missing	6	20%	8	25.8%	14	23%
No	21	70%	21	67.7%	42	68.9%
Yes	3	10%	2	6.5%	5	8.2%
**Condition worsened**						
Missing	5	16.7%	8	25.8%	13	21.3%
No	22	73.3%	21	67.7%	43	70.5%
Yes	3	10%	2	6.5%	5	8.2%
**6 Months**						
**Recent falls (last 3 months)**						
No	23	76.7%	25	80.6%	48	78.7%
Yes	3	10%	2	6.5%	5	8.2%
Missing	4	13.3%	4	12.9%	8	13.1%
**Number of recent falls**						
0	27	90%	29	93.5%	56	91.8%
1	2	6.7%	1	3.2%	3	4.9%
2	1	3.3%	0	0%	1	1.6%
5	0	0%	1	3.2%	1	1.6%
**Broken bones (from recent fall)**						
Yes	1	3.3%	0	0%	1	1.6%
No	2	6.7%	2	6.5%	4	6.6%
**Pain increase**						
Missing	3	10%	5	16.1%	8	13.1%
No	27	90%	26	83.9%	53	86.9%
Yes	0	0%	0	0%	0	0%
**Condition worsened**						
Missing	3	10%	6	19.4%	9	14.8%
No	26	86.7%	25	80.6%	51	83.6%
Yes	1	3.3%	0	0%	1	1.6%

For peer review only

For peer review only

CONSORT 2010 checklist of information to include when reporting a pilot or feasibility trial*

Section/Topic	Item No	Checklist item	Reported on page No
**Title and abstract**			
	1a	Identification as a pilot or feasibility randomised trial in the title	1
	1b	Structured summary of pilot trial design, methods, results, and conclusions (for specific guidance see CONSORT abstract extension for pilot trials)	2
**Introduction**			
Background and objectives	2a	Scientific background and explanation of rationale for future definitive trial, and reasons for randomised pilot trial	4-5
	2b	Specific objectives or research questions for pilot trial	5
**Methods**			
Trial design	3a	Description of pilot trial design (such as parallel, factorial) including allocation ratio	5
	3b	Important changes to methods after pilot trial commencement (such as eligibility criteria), with reasons	7
Participants	4a	Eligibility criteria for participants	6
	4b	Settings and locations where the data were collected	5
	4c	How participants were identified and consented	5
Interventions	5	The interventions for each group with sufficient details to allow replication, including how and when they were actually administered	7-8, Table 1
Outcomes	6a	Completely defined prespecified assessments or measurements to address each pilot trial objective specified in 2b, including how and when they were assessed	9
	6b	Any changes to pilot trial assessments or measurements after the pilot trial commenced, with reasons	N/A
	6c	If applicable, prespecified criteria used to judge whether, or how, to proceed with future definitive trial	9
Sample size	7a	Rationale for numbers in the pilot trial	11
	7b	When applicable, explanation of any interim analyses and stopping guidelines	N/A
Randomisation:			
Sequence generation	8a	Method used to generate the random allocation sequence	6
	8b	Type of randomisation(s); details of any restriction (such as blocking and block size)	6
Allocation concealment mechanism	9	Mechanism used to implement the random allocation sequence (such as sequentially numbered containers), describing any steps taken to conceal the sequence until interventions were assigned	6

For peer review only

Implementation	10	Who generated the random allocation sequence, who enrolled participants, and who assigned participants to interventions	6
Blinding	11a	If done, who was blinded after assignment to interventions (for example, participants, care providers, those assessing outcomes) and how	6
	11b	If relevant, description of the similarity of interventions	7-8
Statistical methods	12	Methods used to address each pilot trial objective whether qualitative or quantitative	9
**Results**			
Participant flow (a diagram is strongly recommended)	13a	For each group, the numbers of participants who were approached and/or assessed for eligibility, randomly assigned, received intended treatment, and were assessed for each objective	12
	13b	For each group, losses and exclusions after randomisation, together with reasons	Figure 1
Recruitment	14a	Dates defining the periods of recruitment and follow-up	12
	14b	Why the pilot trial ended or was stopped	12
Baseline data	15	A table showing baseline demographic and clinical characteristics for each group	Table 2
Numbers analysed	16	For each objective, number of participants (denominator) included in each analysis. If relevant, these numbers should be by randomised group	12-14
Outcomes and estimation	17	For each objective, results including expressions of uncertainty (such as 95% confidence interval) for any estimates. If relevant, these results should be by randomised group	12-14
Ancillary analyses	18	Results of any other analyses performed that could be used to inform the future definitive trial	12-14
Harms	19	All important harms or unintended effects in each group (for specific guidance see CONSORT for harms)	14
	19a	If relevant, other important unintended consequences	N/A
**Discussion**			
Limitations	20	Pilot trial limitations, addressing sources of potential bias and remaining uncertainty about feasibility	16
Generalisability	21	Generalisability (applicability) of pilot trial methods and findings to future definitive trial and other studies	15-16
Interpretation	22	Interpretation consistent with pilot trial objectives and findings, balancing potential benefits and harms, and considering other relevant evidence	15-16
	22a	Implications for progression from pilot to future definitive trial, including any proposed amendments	16-17
**Other information**			
Registration	23	Registration number for pilot trial and name of trial registry	3
Protocol	24	Where the pilot trial protocol can be accessed, if available	Cited p.5
Funding	25	Sources of funding and other support (such as supply of drugs), role of funders	18
	26	Ethical approval or approval by research review committee, confirmed with reference number	3, 12

For peer review only

Citation: Eldridge SM, Chan CL, Campbell MJ, Bond CM, Hopewell S, Thabane L, et al. CONSORT 2010 statement: extension to randomised pilot and feasibility trials. BMJ. 2016;355.

*We strongly recommend reading this statement in conjunction with the CONSORT 2010, extension to randomised pilot and feasibility trials, Explanation and Elaboration for important clarifications on all the items. If relevant, we also recommend reading CONSORT extensions for cluster randomised trials, non-inferiority and equivalence trials, non-pharmacological treatments, herbal interventions, and pragmatic trials. Additional extensions are forthcoming: for those and for up to date references relevant to this checklist, see www.consort-statement.org.

For peer review only
